# Multiple analytical perspectives of mitochondrial genes in the context of preeclampsia: potential diagnostic markers

**DOI:** 10.3389/fimmu.2025.1595706

**Published:** 2025-07-17

**Authors:** Can Li, Fang Liu, Chao Li, Xiangzhong Zhao, Qiulan Lv, Aiping Jiang, Shuping Zhao

**Affiliations:** ^1^ Department of Obstetrics and Gynecology, Qingdao Women and Children’s Hospital, Shandong University, Jinan, Shandong, China; ^2^ Department of Obstetrics and Gynecology, The Affiliated Hospital of Qingdao University, Qingdao, Shandong, China; ^3^ Department of Obstetrics and Gynecology, Dazhou Dachuan District People’s Hospital (Dazhou Third People’s Hospital), Dazhou, Sichuan, China; ^4^ Department of Medical Research Center, The Affiliated Hospital of Qingdao University, Qingdao, Shandong, China

**Keywords:** preeclampsia, mitochondria-related genes, diagnostic model, machine learning, immune cells infiltration

## Abstract

Preeclampsia(PE) is closely linked to adverse maternal and fetal outcomes. Given the pivotal roles of mitochondria in various human diseases and the limited research on their involvement in PE, this study identified biomarkers linked to mitochondrial metabolism in PE and their roles in its pathogenesis. Data from three datasets were integrated using the ComBat algorithm to mitigate batch effects. Differential expression analysis identified genes differentially expressed between PE cases and Control group. Cross-referencing these genes with mitochondrial energy metabolism-related genes (MMRGs) isolated mitochondrial energy metabolism-related differentially expressed genes (MMRDEGs). GO and KEGG analysis were performed to elucidate the functions of the MMRDEGs. A diagnostic model using Random Forest and logistic regression was validated by ROC curve analysis. mRNA expressions of *OCRL*, *TPI1*, *GAPDH*, and *LDHA* were quantified via qPCR. Immune characteristics were explored, and PPI, mRNA-miRNA, mRNA-TF and mRNA-RBP interaction networks were constructed. AlphaFold analyzed protein structures of *OCRL, TPI1, GAPDH*, and *LDHA.* A total of 1073 DEGs and 24 MMRDEGs were identified. *OCRL, TPI1, GAPDH*, and *LDHA* formed the diagnostic model, which were predominantly enriched in pyruvate metabolism, glycolysis, and ATP metabolism pathways. CIBERSORT highlighted immune cell composition variations between PE and Control groups. *OCRL, TPI1, GAPDH*, and *LDHA* exhibited increased mRNA expression levels in preeclamptic placentas. Therefore, MMRDEGs may play a critical role in the mechanism of oxidative stress and inflammatory response in PE by mediating metabolic regulation and immune modulation, potentially serving as diagnostic biomarkers associated with mitochondrial metabolism in preeclampsia.

## 1 Introduction

Preeclampsia (PE) is a pregnancy-related multisystem syndrome that occurs at or after 20 week of gestation, characterized by elevated blood pressure (systolic blood pressure ≥140 mmHg and/or diastolic blood pressure ≥90 mmHg) and proteinuria (≥300 mg/24h). This condition can lead to multiple organ dysfunctions, including hematological abnormalities, hepatic impairment, and renal insufficiency. In severe cases, it may also compromise pulmonary function, retinal health, and the integrity of the central nervous system (1–4). PE is one of the leading causes of maternal mortality globally, with an estimated prevalence of approximately 10% (5). Its pathogenesis is closely associated with placental vascular insufficiency, endothelial dysfunction, heightened inflammatory responses, immune imbalance, and systemic small-vessel spasms (6–8). Currently, the management of PE primarily relies on blood pressure control and timely pregnancy termination (9, 10). However, the limited availability of preventive and intervention strategies leads to a high incidence of iatrogenic preterm birth, thus increasing the risk of adverse perinatal outcomes. Therefore, an in-depth exploration of the pathogenesis of PE is essential for reducing its incidence and improving prognostic outcomes.

Mitochondria play a pivotal role in cellular bio-oxidation and energy metabolism, being involved in a range of physiological processes including biosynthesis and signal transduction (11, 12). Therefore, mitochondrial dysfunction disrupts these processes, resulting in elevated generation of reactive oxygen species (ROS) and enhanced apoptosis (13–15). As a critical organ for maternal-fetal material exchange, synthesis, defense, and immunity, the placenta exhibits a high demand for energy, primarily supplied by ATP generated by mitochondria (16, 17). If mitochondrial function diminished, ATP synthesis will consequently decrease, thereby impairing placental function and increasing the risk of complications such as preeclampsia (PE), gestational diabetes mellitus(GDM), and fetal growth restriction(FGR) (18, 19). Numerous studies have demonstrated that elevated levels of oxidative stress in patients with PE contribute to the promotion of inflammatory responses and mitochondrial dysfunction (20, 21). Research further suggests that mitochondrial dysfunction plays a critical role in both the onset and progression of PE. For instance, Long et al. (22)reported that mitochondrial damage leads to trophoblast dysfunction, which in turn contributes to the pathogenesis of PE. These findings suggest that targeting mitochondrial repair could represent a promising therapeutic strategy for managing this condition. In addition, several mitochondria-associated genes, such as *CPOX*, *DEGS1*, and *SH3BP5*, have been validated to possess significant diagnostic value for PE (18). Mitochondria harbor an independent genome distinct from nuclear DNA (23), and alterations in the expression of specific mitochondrial genes have been identified as being closely linked to the diagnosis and treatment of PE (24, 25). In recent years, accumulating evidence has demonstrated that immune cell infiltration is a critical factor in the pathogenesis of various diseases, including PE, preterm birth, GDM, and osteoarthritis (26–28).

Owing to the multifaceted nature of PE, there has been relatively limited progress in its prediction and prevention (29). Given the pivotal role of mitochondrial energy metabolism in various diseases, further exploration into the mechanisms of mitochondrial energy metabolism in PE carries significant clinical implications. This study is expected to offer a theoretical basis and innovative perspectives for the early diagnosis and therapeutic intervention of PE. In this study, we aimed to utilize machine learning techniques to construct an innovative diagnostic model for PE and investigate the association between key differentially expressed genes (DEGs) and immune infiltration. Additionally, we validated the expression levels of these DEGs in placental tissues from PE patients, thereby highlighting their potential significance in the pathophysiological mechanisms underlying PE.

## 2 Materials and methodologies

### 2.1 Sample collection

In this research, 20 PE placental tissues, among which 12 cases with severe features, were collected following cesarean sections, with diagnosis conforming to the guidelines established by the Task Force on Hypertension in Pregnancy. Correspondingly, control placental tissues (n=20), matched for age and body mass index (BMI), were also obtained. All placental tissues were sourced from pregnant women who delivered at the Affiliated Hospital of Qingdao University. Because gestational diabetes mellitus (GDM) is associated with an increased incidence of PE (30), exclusion criteria for the research were as follows: twin or multiple pregnancies; fetal structural abnormalities or chromosomal anomalies; the presence of comorbidities or complications including GDM, pre-pregnancy diabetes mellitus, chronic hypertension, cardiac, renal, or liver diseases, infectious diseases, or autoimmune disorders; history of blood transfusion, organ transplantation, or immunotherapy; and any history of smoking, alcohol consumption, or substance abuse. Basic clinical data were collected for this study, encompassing age, BMI, gestational week at delivery, parity, systolic and diastolic blood pressure, newborn weight, and one-minute Apgar score. A sample of maternal placental tissue, approximately 1 cm in diameter, was collected within ten minutes of placental delivery, specifically avoiding areas with infarcts or calcification. These samples were then placed in freezing tubes containing RNA preservation solution and kept at -80°C for subsequent analysis using RT-qPCR. All the participants of the study provided a written informed consent. The investigation was approved by the Ethics Committee of the Affiliated Hospital of Qingdao University (Approval No: QYFY WZLL 28705). It was carried out in rigorous adherence to the guidelines established by the committee.

### 2.2 Data download

The expression profile datasets GSE24129 (31), GSE30186 (32), GSE54618 (33) and GSE75010 (34) of patients with PE were obtained from GEO database (35) utilizing the GEOquery package (36). The GSE24129 dataset included 16 placental samples, evenly distributed between 8 PE cases and 8 Control group. The GSE30186 dataset comprised 12 placental samples, with an equal number of PE cases and Control group. The GSE54618 dataset consisted of 17 placental samples, including 5 from PE cases and 12 from Control group. Lastly, the GSE75010 dataset contained 80 PE cases and 77 Control group. The dataset GSE24129 and GSE75010 utilized the GPL6244 [HuGene-1_0-st-v1] Affymetrix Human Gene 1.0 ST Array [transcript (gene) version]. For the datasets GSE30186 and GSE54618, the associated platform was the GPL10558 Illumina HumanHT-12 V4.0 expression beadchip. The microarray GPL platform files facilitated related annotation for the probe names across these datasets. Specific information for each dataset is depicted in **Supplementary Table 1**. Utilizing “mitochondrial energy metabolism” as the search keyword and focusing solely on protein-coding genes, we extracted 219 MMRGs from the datebase of GeneCards (https://www.genecards.org/),which offers extensive data on the human genes (37). Additionally, we derived 188 MMRGs from the published literature (38). By integrating these datasets and removing duplicates, we compiled a consolidated list of 384 MMRGs. The specific names of these genes are listed in **Supplementary Table 2**.

### 2.3 Preprocessing the datasets and differential expression analysis

We integrated the GSE24129, GSE30186 and GSE54618 datasets and then eliminated batch effects by applying the ComBat algorithm from the R package (39), followed by normalization using the normalize Between Arrays function. Thus, the Combined dataset (including 19 PE cases and 26 Control group) was obtained. Subsequently, we obtained DEGs by utilizing R’s limma package to carry out a differential analysis of the expression of all genes among the PE and the control cohort samples of the combined dataset. To make sure to capture all changes in expression levels, whether up-regulated or down-regulated, we made a screening standard of p < 0.05 plus | logFC | > 0 to further study the DEGs (40). The findings of variance analysis through the R package ggplot2 map volcano to display. Then, we took MMRGs and DEGs intersection to obtain the MMRDEGs.

### 2.4 GO and KEGG analysis

The GO (41) approach is frequently employed in large-scale functional enrichment investigations for the purpose of categorizing genes into groups that are associated with biological process (BP), molecular function (MF), and cellular component (CC). The KEGG (42) serves as a crucial repository for genomic information, diseases, drug-related data and biological pathways. We conducted GO and KEGG annotation analyses of MMRDEGs by employing the R package clusterProfiler (43). We set a marked threshold (*p < 0.05*) for pathway selection, ensuring that only statistically significant pathways were considered in our analysis.

### 2.5 GSEA and GSVA analysis

GSEA (44) is a widely utilized method for assessing variations in pathway activity and biological process involvement across different sample groups within an expression dataset. In this research, we initially carried out a differential gene expression analysis between various groups (PE/Control and High/Low Risk score) within the combined dataset. Subsequently, genes were categorized into two cohorts based on their logFC values: those with positive and those with negative logFC. For the enrichment analysis of these categorized genes, we utilized the clusterProfiler package. The GSEA configuration for this analysis utilized the following specifications: a seed of 2022, 1000 permutations, and a gene set size ranging from a minimum of 10 to a maximum of 500 genes. We retrieved the gene set “c2.Cp.All.V2022.1.Hs.Symbols.GMT [All Canonical Pathways]” containing 3050 entries from the Molecular Signatures Database (MSigDB) (45). Pathways which got a significant enrichment level (*p < 0.05)* were deemed markedly enriched.

GSVA (46) was designed to assess gene set enrichment within microarray and nuclear transcriptome data. This technique enables the conversion from diverse samples into a sample-specific gene expression matrix and can evaluate the pathway enrichment across multiple specimens. In this study, we also employed the gene set used earlier when GSEA analysis was performed. GSVA was carried out on gene expression matrices derived from distinct groups (PE/Control or High/Low Risk score) within the Combined dataset, utilizing this reference gene set. The analysis revealed functional disparities in enriched pathways between sample cohorts within the Combined dataset. Pathways with a significance level (*p < 0.05*) were further scrutinized; specifically, we selected and examined the 10 pathways exhibiting both the largest and smallest log fold change (logFC).

### 2.6 Construct MMRDEGs diagnostic model

The RandomForest (RF) (47) technique is a collective learning approach that integrates numerous decision tree models. It belongs to the bagging (bootstrap aggregation) ensemble algorithm, which consists of multiple algorithms. RF is a commonly used approach for model building. By constructing multiple decision trees, the prediction results of each tree in the forest are aggregated using a voting method to obtain the final prediction result for a given sample. In this study, we utilized the MMRDEGs expression levels in the Combined dataset’s expression matrix to build a model using the RandomForest package with parameter set.seed (2023) and ntree = 1000.


$$I\left(X=xi\right)=\;-log_{2}p\left(x_{i}\right)$$

We conducted a logistic regression analysis on MMRDEGs to construct a Logistic diagnostic model of the Combined dataset. Moreover, we employed the Logistic regression to analyze the association of the independent variables and dependent variables, when considering dependent variables as binary variables(PE cases and Control group). *p* < 0.05 was a significance level as criteria for identifying MMRDEGs and constructing the Logistic diagnostic model. The molecular expressions of MMRDEGs incorporated in this logistic regression model were visualized through Forest Plot.

Furthermore, we conducted the Least Absolute Shrinkage and Selection Operator (LASSO, the seed number is 2022) by R package glmnet (48) to process the MMRDEGs, which were screened out by utilizing our logistic regression model, to obtain the Logistic-LASSO regression model. LASSO regression analysis reduces overfitting incorporating a penalization factor (lambda × absolute value of slope), thereby improving its capacity for generalization while maintaining interpretability. The results obtained from LASSO analysis were depicted through variable trajectory plot techniques and diagnostic model plot.


$$\text{risk}Score\;=\;\sum_{i}Coefficient\;\left(hub\;gene_{i}\right)*mRNA\;Expression\;\left(hub\;gene_{i}\right)$$

Subsequently, we identified the common MMRDEGs by intersecting the MMRDEGs derived from both the RF model and the Logistic-LASSO regression model, which were then visualized using a Venn diagram. The expression levels of the common MMRDEGs in the Combined dataset were combined with the coefficients of these genes in the regression model of Logistic-LASSO to establish an MMRDEGs diagnostic model and to calculate corresponding Risk-scores. A Nomogram (49), a visual depiction of interrelations among several independent variables on a rectangular plane-coordinate system, was constructed based on the gene expression levels derived from the MMRDEGs diagnostic model generated through Logistic LASSO regression analysis in the Combined dataset. To examine the precision and distinguishing capability of our MMRDEGs diagnostic models, Decision Curve Analysis (DCA) (50), a straightforward approach for appraising molecular markers, diagnostic tests and clinical prediction models, was performed using the ggDCA R package.

### 2.7 Analysis of the infiltration of immune cells

The relative abundance of a variety of immune cell infiltrates within every sample was quantified utilizing the single-sample gene-set enrichment analysis (ssGSEA) algorithm. The method facilitated to label various immune cell types. For instance, regulatory T cells, CD8^+^ T cells, dendritic cells and macrophages. We represented the relative abundance of each immune cell type across the samples by enrichment scores, which were calculated utilizing ssGSEA methodology (51, 52). Using the ssGSEA algorithm from the GSVA R package (version 1.46.0), we calculated the enrichment scores of groups within high and low risk cohorts according to the MMRDEGs diagnostic model from the Combined dataset. These scores depicted the extent of immune cell infiltrations in individual specimen, thereby illustrating disparities of the abundance of immune cell infiltration among the different (High and Low) risk cohorts through box plots. Additionally, we examined the correlation of immune cell abundances among the high and low risk cohorts utilizing scatter plots. The association among immune cells and commonly altered MMRDEGs across these groups was analyzed using the Spearman statistical method and depicted in correlation dot plots, increasing our understandings of the immune landscape in relation to preeclampsia risk stratification.

CIBERSORT (53) is a kind of immune infiltration algorithm, that deconvoluted transcriptome expression matrices based on linear support vector regression, to assess the abundance and composition of immune cells within different samples. For this analysis, we input the expression matrix data of samples of the High and the Low risk groups defined by the MMRDEGs diagnostic model in the Combined dataset to CIBERSORT. Using the feature gene matrix of LM22, we refined the results by retaining solely those data points with immune cell enrichment scores >0, thus obtaining and visualizing the comprehensive findings of the immune cell infiltration abundance matrix. Those disparities in immune cell infiltration between the high risk and low risk cohorts were depicted utilizing stacked bar charts. We employed the Spearman statistical method to analyze the correlations among immune cells within the Combined dataset and utilized the R package ggplot2 to visualize the results. Moreover, the interactions among immune cells and commonly altered MMRDEGs were depicted using correlation dot plots, providing insights into the immune dynamics associated with different risk stratifications in PE.

### 2.8 PPI network and mRNA-RBP, mRNA-TF, mRNA-Drug interaction network

The protein-protein interaction (PPI) network consists of individual proteins that engage with one another. In this study, we constructed the common MMRDEGs PPI network(minimum required interaction score: low confidence (0.150)) using the database of STRING (54). The network was visualized using Cytoscape, which allowed us to identify densely interconnected clusters within the PPI network. These clusters potentially signify molecular assemblies with unique biological roles, offering insights into the molecular mechanisms underlying PE.

ENCORI database (55) (https://starbase.sysu.edu.cn/) facilitates the exploration of interactions among various RNA types, including microRNAs-ncRNA, microRNAs-mRNA, ncRNA-RNA, and RNA-RNA, as well as interactions among RNA-binding proteins (RBPs) and ncRNAs or mRNAs. These interactions are curated utilizing degradome sequencing data and CLIP-seq, supporting comprehensive visual tools of investigating miRNA targets. In our study, we utilized the ENCORI database to forecast RBPs interacting with the commonly altered MMRDEGs. We established “pancancerNum> 27” as the threshold for selecting significant interactions. and the mRNA-RBP interaction network was rendered utilizing Cytoscape.

HTFtarget database (56) (http://bioinfo.life.hust.edu.cn/hTFtarget) integrates human transcription factors (TFs) and their corresponding control targets data. The CHIPBase database (https://rna.sysu.edu.cn/chipbase/) predicted transcriptional regulatory relationships among millions of TFs and genes. Utilizing both HTFtarget databases and CHIPBase, the TFs that link to common MMRDEGs were identified. We applied the screening criteria of having an upstream and downstream sample count greater than zero. Subsequently, the mRNA-TF interactive network was rendered visually utilizing Cytoscape software.

### 2.9 RT-qPCR

Placental tissues were lysed using FreeZol reagent (Vazyme, R711) following the manufacturer’s instructions. RNA concentration and purity were measured using a spectrophotometer. The isolated RNA was reverse transcribed into cDNA with a reverse transcription kit (Agbio, AG11705). Real-time polymerase chain reaction (qPCR) was then performed using the SYBR Green Pre-Mix Pro Taq HS qPCR Kit (Agbio, AG11701). Relative gene expression levels were normalized to *β-actin* and calculated using the 2^^(-ΔΔCt)^ method. The **Supplementary Table 3** lists the primer sequences of mRNAs and internal control.

### 2.10 Statistical analysis

The entirety of data manipulation and statistical evaluation in this investigation was executed utilizing R software (Release 4.1.2). The independent Student’s t-test was used to compare continuous variables (fit normal distribution). We employed the Mann-Whitney U test (Wilcoxon rank sum test) for variables lacking normally distributed. Use Spearman correlation analysis to computer the findings unless otherwise specified. The p-values for statistical tests are two-tailed, and a threshold of 0.05 is deemed indicative of statistically meaningful results.

## 3 Results

### 3.1 Dataset processing

**Figure 1:**
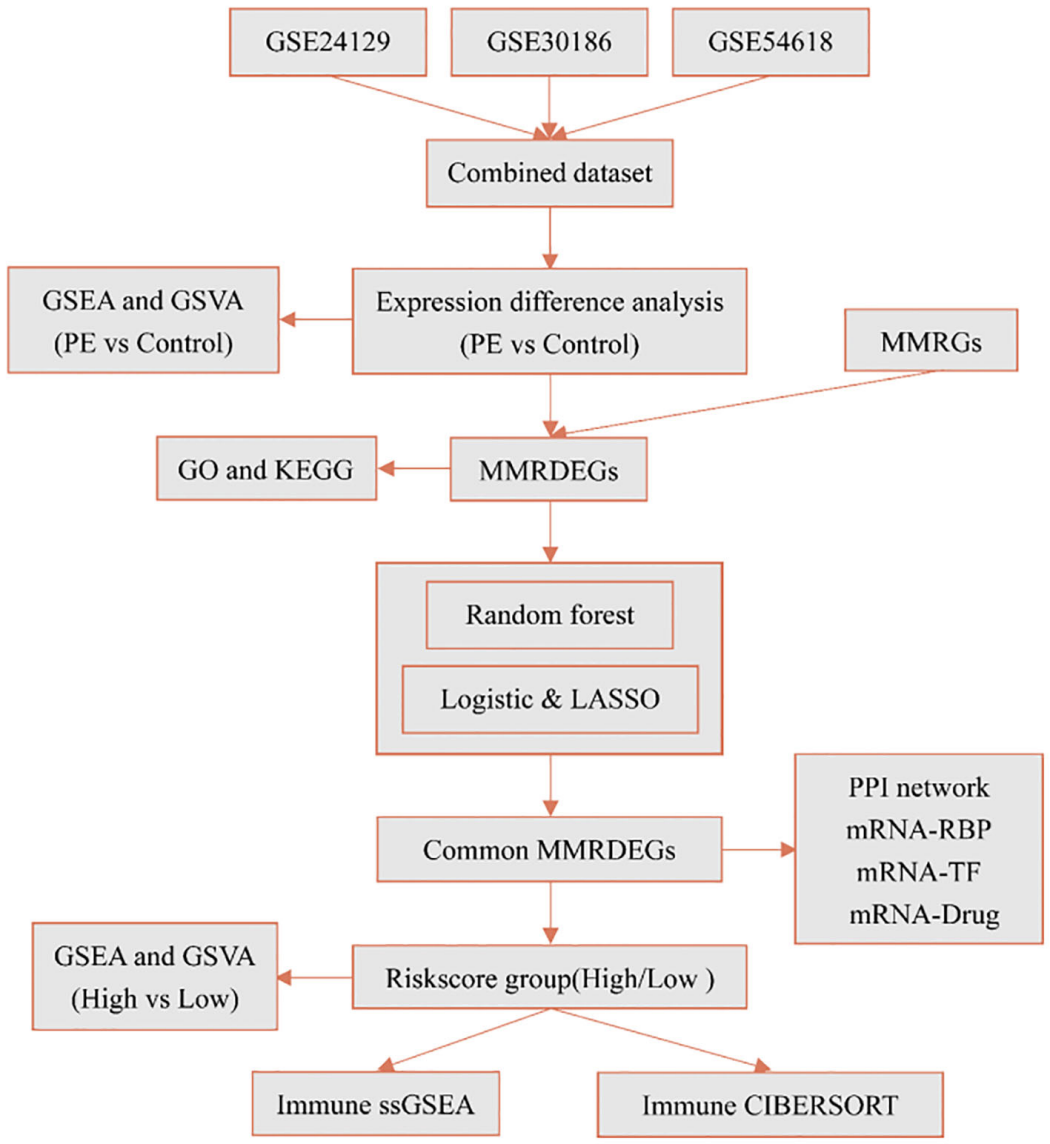
Technology roadmap. PE, Preeclampsia; GSEA, Gene Set Enrichment Analysis; GSVA, Gene Set Variation Analysis; MMRGs, Mitochondrial energy metabolism-related genes; MMRDEGs, Mitochondrial energy metabolism related differentially expressed genes; GO, Gene Ontology; KEGG, Kyoto Encyclopedia of Genes and Genomes; LASSO, Least absolute shrinkage and selection operator; RBP, RNA binding protein; TF, Transcription factors; ssGSEA, single-sample gene-set enrichment analysis.

According to the technical roadmap of this experiment (**Figure 1**), we first combined the three datasets (GSE24129, GSE30186, and GSE54618), then batched the data using the ComBat function from R’s sva package, and then utilized the Normalize Between Arrays function of the limma package to perform standardization procedures. The dataset of 19 PE cases and 26 Control group, that was combined, was obtained.

The before and after data processing boxplots and PCA plots of the combined dataset, according to the sample source, were showed in the **Supplementary Figures 1A–D**, respectively. The findings demonstrated that the expression profiles of samples from the Combined dataset exhibited a high degree of consistency, indicating successful removal of batch effects through data processing. The Combined dataset utilized for subsequent analyses represented the batch effect-corrected and normalized data.

We further utilized the limma package to standardize the GSE75010 dataset and compared the pre- and post-processing states of the dataset using boxplots (**Supplementary Figures 1E, F**). The boxplot analysis demonstrated that the expression levels of samples in the GSE75010 dataset became significantly more consistent after data processing.

### 3.2 Combined dataset differential expression analysis of PE and control groups

The placenta serves as a critical organ facilitating material transport between mother and fetus, performing multiple functions during pregnancy such as immune protection, endocrine regulation, and serving as a conduit for nutrient and oxygen delivery. Its condition is closely associated with the health of both mother and child during gestation. Torbergsen T et al. first described a high incidence of preeclampsia in a family with mitochondrial disorder (57). Recent research into the mechanisms underlying preeclampsia has revealed mitochondrial dysfunction in both patients with preeclampsia and animal models (58).

**Figure 2:**
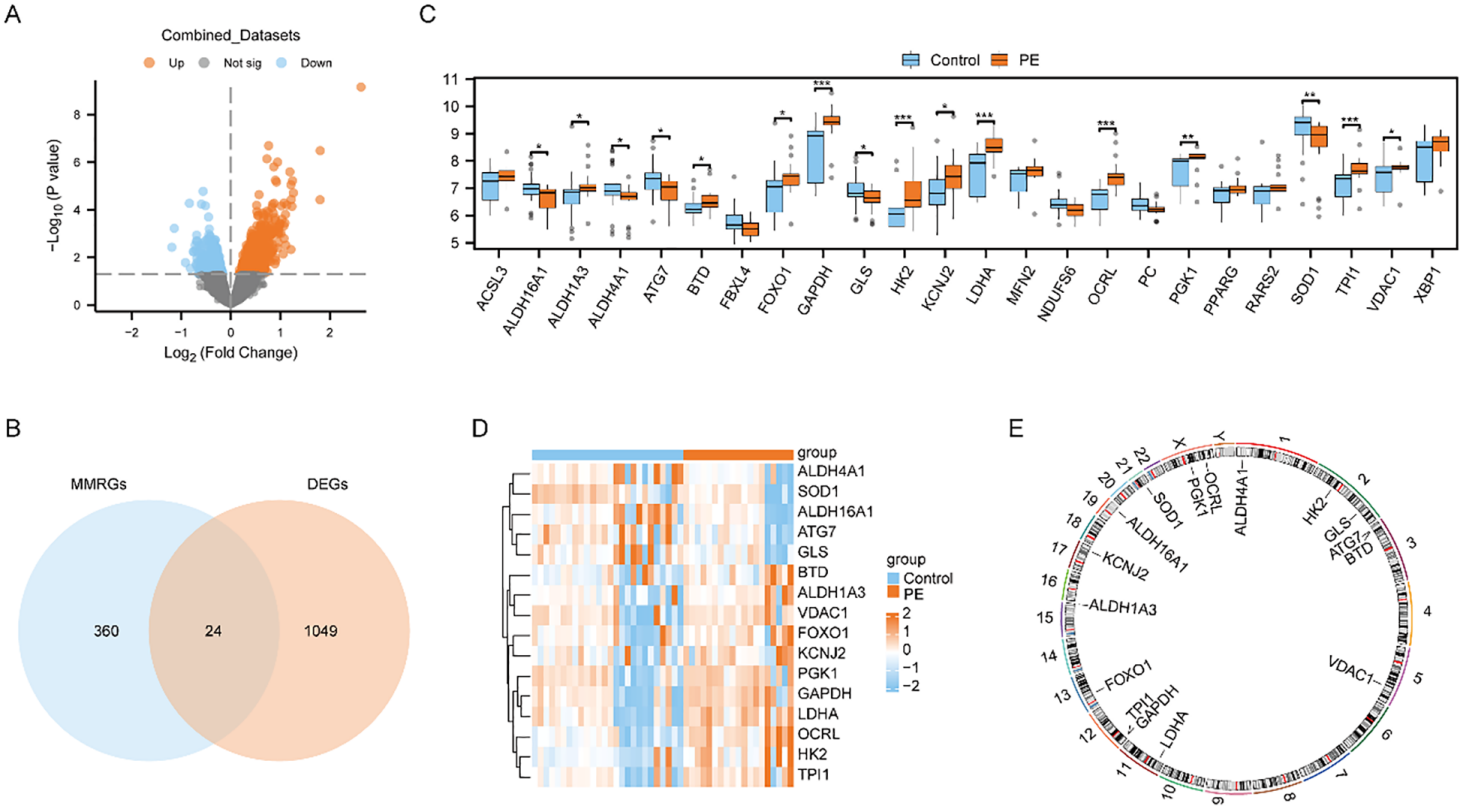
Differential expression analysis and correlation analysis of MMRDEGs. **(A)** Volcano plot presentation of the results of differential analysis between PE cases and Control group in Combined datasets. **(B)** Venn diagram of DEGs between PE cases and Control group and MMRGs in Combined datasets. **(C)** Group comparison plot of MMRDEGs between PE cases and Control group in Combined datasets. **(D)** Simplified numerical heatmap of MMRDEGs in Combined datasets. **(E)** Chromosomal mapping of MMRDEGs. The symbol ns was equivalent to *p ≥ 0.05*, which was not statistically significant. The symbol * is equivalent to *p < 0.05*, which is statistically significant; The symbol ** is equivalent to *p < 0.01*, which is highly statistically significant; The symbol *** is equivalent to *p < 0.001* and highly statistically significant. PE, Preeclampsia; DEGs, differentially expressed genes; MMRGs, Mitochondrial energy metabolism related genes; MMRDEGs, Mitochondrial energy metabolism related differentially expressed genes.

We utilized limma package to explore the Combined dataset of PE cases and Control group. And we got 1073 differentially expressed genes using the threshold of | logFC | > 0 and *p* < 0.05, including 603 highly expressed genes in PE cases(the Control group of samples low expressed, logFC is positive, raised genes), and 470 genes low expressed in PE cases(the Control group of samples increased, logFC is negative, cut genes). And then, we presented the outcomes of differential expression analysis between the two groups in the Combined dataset using the volcano plot (**Figure 2A**). We then intersected these 1073 differently expressed genes (DEGs) with 384 mitochondrial energy metabolism-related genes (MMRGs). And then, 24 mitochondrial energy metabolism-related differentially expressed genes (MMRDEGs) were identified. The 24 MMRDEGs were *ACSL3*, *ALDH16A1*, *ALDH1A3*, *ALDH4A1*, *ATG7*, *BTD*, *FBXL4*, *FOXO1*, *GAPDH*, *GLS*, *HK2*, *KCNJ2*, *LDHA*, *MFN2*, *NDUFS6*, *OCRL*, *PC*, *PGK1*, *PPARG*, *RARS2*, *SOD1*, *TPI1*, *VDAC1*, *XBP1* (**Figure 2B**).

We also generated a comparative map to analyze the differential expression of 24 MMRDEGs between the two cohorts (**Figure 2C**). The analysis suggested that 16 MMRDEGs exhibited significant differences, with *ALDH16A1*, *ALDH4A1*, *ATG7*, *GLS* and *SOD1* significantly down-regulated while *ALDH1A3*, *BTD*, *FOXO1*, *GAPDH*, *HK2*, *KCNJ2*, *LDHA*, *OCRL*, *PGK1*, *TPI1*, and *VDAC1* significantly up-regulated.

Then, we drew a simple numerical heat map derived from the expression matrix of these 16 MMRDEGs above (**Figure 2D**), and the visualization revealed substantial disparities in the expression patterns of the 16 MMRDEGs between the two sample groups. Additionally, we annotated the positions of these 16 MMRDEGs and draw a chromosome localization map (**Figure 2E**) by employing the RCircos package, from which the specific distribution of the 16 MMRDEGs on each chromosome can be obtained.

### 3.3 The GO and the KEGG analysis of MMRDEGs

**Figure 3:**
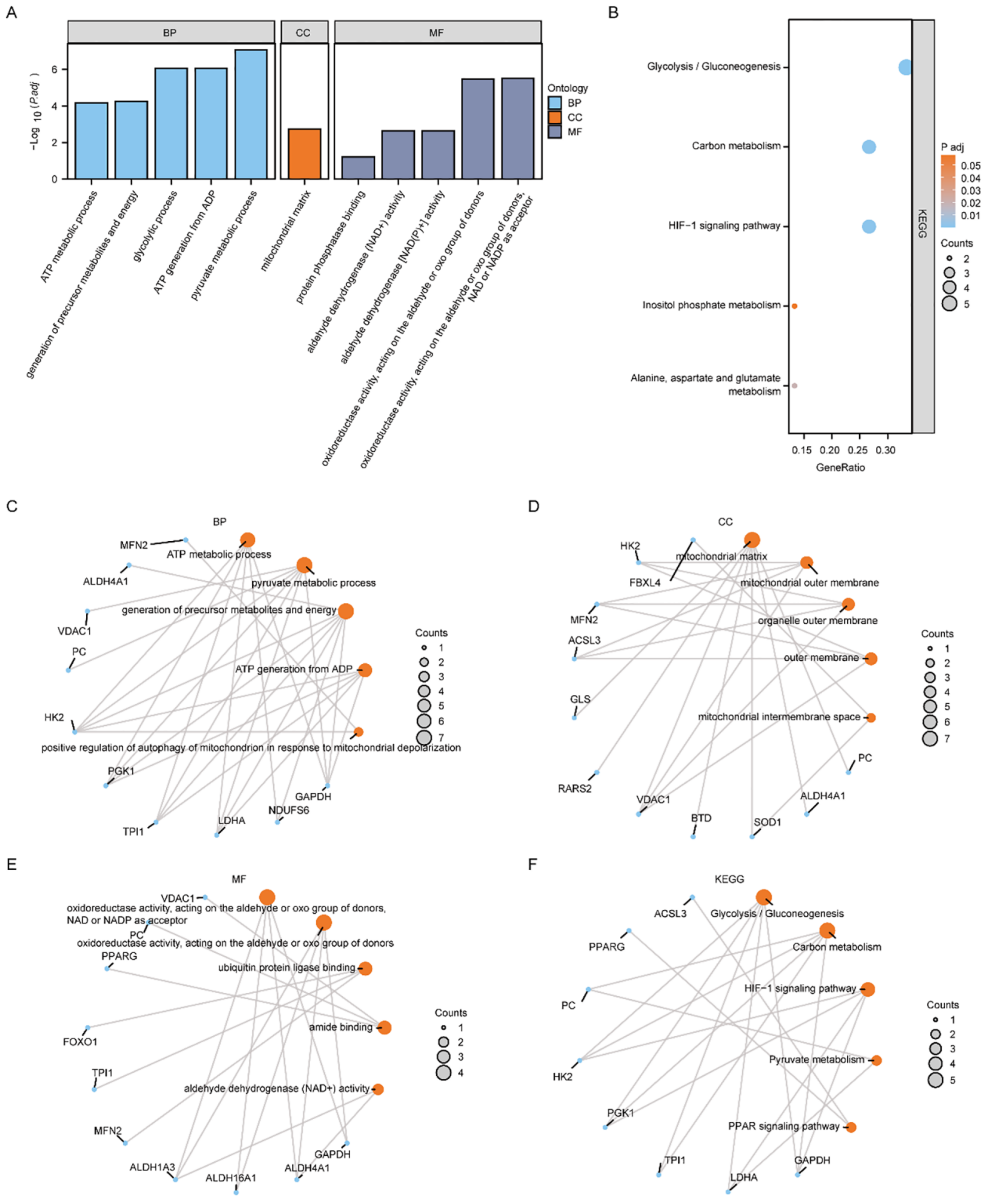
Functional enrichment analysis (GO) and pathway enrichment (KEGG) analysis of MMRDEGs. **(A)** Bar graph showing the GO enrichment analysis results of MMRDEGs. **(B)** Bubble plot display of KEGG pathway enrichment analysis results of MMRDEGs. **(C–F)** Loop network diagram of BP pathway **(C)**, CC pathway **(D)**, MF pathway **(E)** and KEGG pathway **(F)** in MMRDEGs enrichment analysis results. In the bar graph **(A)**, the abscissa is the GO terms, and the height of the bar indicates the Padj value of GO terms. In the network diagram **(C–F)**, blue dots represent specific genes, and orange dots represent specific pathways. MMRDEGs, Mitochondrial energy metabolism related differentially expressed genes; GO, Gene Ontology; BP, biological process; CC, cellular component; MF, molecular function; KEGG, Kyoto Encyclopedia of Genes and Genomes; The screening criterion for GO/KEGG enrichment items was *p < 0.05*.

The biological processes (BP), molecular functions (MF), cellular components (CC), relationships between biological pathways, pathway enrichment analysis using the Kyoto Encyclopedia of Genes and Genomes (KEGG), and gene function enrichment analysis based on Gene Oncology (GO) were carried out to analyze the 16 MMRDEGs. Pathways that below the *P* threshold of 0.05, were considered to be statistically significant. The outcomes showed that the 16 MMRDEGs main enriched in those BPs, such as pyruvate metabolic process, glycolytic process, ATP generation from ADP, generation of precursor metabolites and energy, ATP metabolic process. And in the CCs of the mitochondrial matrix. It was enriched in acting on the aldehyde or oxo group of donors, oxidoreductase activity, NAD or NADP as acceptor, aldehyde dehydrogenase (NAD+) activity, aldehyde dehydrogenase [NAD(P)+] activity, protein phosphatase binding, oxidoreductase activity, acting on the CH-NH group of donors, NAD or NADP as acceptor and other MFs (**Figure 3A**). It was also enriched in Glycolysis/Gluconeogenesis, HIF-1 signaling pathway, Carbon metabolism, Alanine, aspartate, glutamate metabolism, Inositol phosphate metabolism (**Figure 3B**) and other KEGG pathways (**Supplementary Table 4**). In addition, the enrichment consequences of the BP pathways (**Figure 3C**), CC pathways (**Figure 3D**), MF pathways (**Figure 3E**), and KEGG pathways (**Figure 3F**) of GO analysis were presented utilizing ring network diagrams.

We utilized the Pathview R package for pathway mapping to illustrate the KEGG enrichment results of Glycolysis/Gluconeogenesis (**Supplementary Figure 2A**), Carbon metabolism (**Supplementary Figure 2B**), Alanine, aspartate and glutamate metabolism (**Supplementary Figure 2C**), Inositol phosphate metabolism (**Supplementary Figure 2D**), and HIF-1 signaling pathway (**Supplementary Figure 2E**).

### 3.4 GSEA enrichment analysis and GSVA analysis of the control and the PE groups based on the Combined dataset

**Figure 4:**
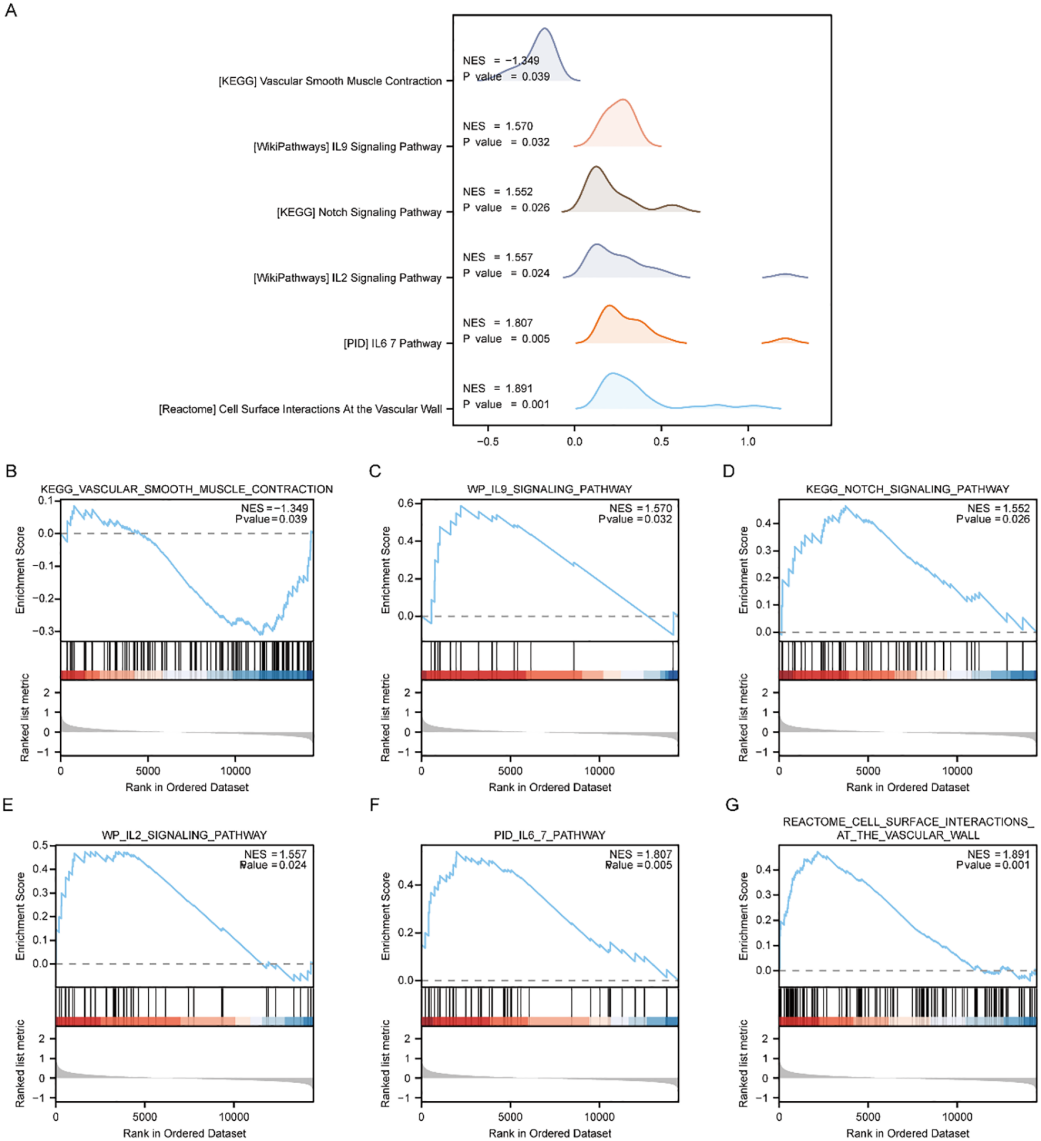
GSEA enrichment analysis between PE cases and Control group in Combined dataset. **(A)** Six main biological characteristics of GSEA enrichment analysis of genes between different groups (PE/Control) of Combined dataset. **(B–G)** Genes in Combined dataset were significantly enriched in KEGG vascular smooth muscle contraction **(B)**, IL9 signaling pathway **(C)**, KEGG NOTCH signaling pathway **(D)**, IL2 signaling pathway **(E)**, IL6/7 pathway **(F)**, Cell surface interactions at the vascular wall **(G)**. PE, Preeclampsia; GSEA, Gene Set Enrichment Analysis. The significant enrichment screening criterion for GSEA enrichment analysis was *p < 0.05*.

To appraise the influence of gene expression levels of genes from PE and Control groups of Combined dataset on PE, we examined the relationships between the expression levels of all genes in different groups (PE/Control) of the Combined dataset and the BPs, CCs, and MFs they played, by employing the Gene Set Enrichment Analysis (GSEA). *p < 0.05* was set as the significant enrichment criterion. The results demonstrated a significant enrichment of genes from different (PE/Control) groups in the Combined dataset, specifically in the vascular smooth muscle contraction pathway (**Figure 4B**), IL9 signaling pathway (**Figure 4C**), Notch signaling pathway (**Figure 4D**), IL2 signaling pathway (**Figure 4E**), IL6/7 signaling pathway (**Figure 4F**), cell surface interactions at the vascular wall (**Figure 4G**), and other pathways (**Supplementary Table 5**). In addition, the outcomes of GSEA analyzing genes between distinct cohorts (PE/Control) of the Combined dataset were depicted by mountain plot (**Figure 4A**).

To investigate the distinctions between disease and controls from the Combined dataset, we then performed Gene Set Variation Analysis (GSVA). From the pathways with *p < 0.05*, we identified 10 pathways with the highest and lowest logFC for further examination (**Supplementary Table 6**), respectively.

The results of GSVA analysis on all the genes of the Combined dataset revealed significant differences among PE and Control groups. Specifically, IKEDA Mir133 targets DN, hyaluronan biosynthesis and export, RHOT1 GTPASE cycle, neurofascin interactions, Irinotecan pathway, Aflatoxin B1 metabolism, Sulindac metabolic pathway, weber methylated LCN in SPERM DN, Tomlins metastasis upregulation of steroid biosynthesis. Additionally, activated NTRK2 signals through FYN and PI3K pathways were observed along with NTRK2 activation of RAC1. Furthermore, HIF1A and PPARG were found to regulate glycolysis. Calvet Rinotecan sensitive vs resistant upregulation was also identified as well as Korkola choriocarcinoma involvement. Lastly erythrocytes demonstrated oxygen uptake and carbon dioxide release while Tesar Alk targeted human es 4D and 5D DN along with JAK targeting mouse es D4 DN. Utilizing the outcomes derived from GSVA, we carried out a differential expression analysis of 20 pathways among PE and control cohorts of the Combined dataset. Subsequently, we created a heatmap illustrating the particular differential analysis outcomes (**Supplementary Figure 3A**) employing the R package. Furthermore, we assessed the extent of group divergence for these 20 pathways across various cohorts from the Combined dataset, utilizing the Mann-Whitney U test, and we use a group comparison plot to illustrate the outcomes (**Supplementary Figure 3B**). The findings demonstrated marked differences in pathway expression among disease control cohorts within the Combined dataset.

### 3.5 Construction of MMRDEGs diagnostic model

**Figure 5:**
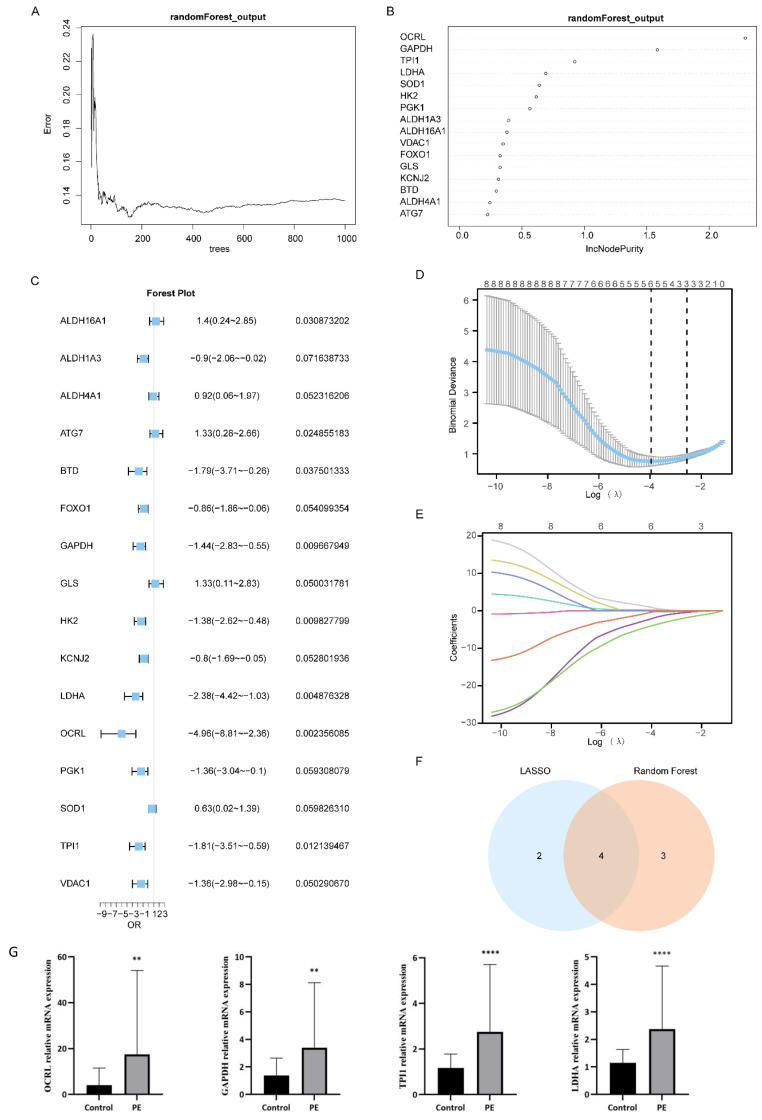
Construction of MMRDEGs diagnostic model. **(A)** Plot of model training error of RF algorithm. **(B)** IncNodePurity presentation of MMRDEGs in the RF model (in descending order of IncNodePurity). **(C)** Forest Plot of Logistic regression model for MMRDEGs. **(D)** Diagnostic model plot of LASSO regression model. **(E)** Variable trajectory plot of LASSO regression model. **(F)** Venn diagram of MMRDEGs in LASSO regression model and MMRDEGs in RF model. **(G)** The mRNA expressions of *OCRL*, *GAPDH*, *TPI1* and *LDHA* of placental tissues in the PE cases and Control group. The symbol ** is equivalent to *p < 0.01*, which is highly statistically significant; The symbol **** is equivalent to *p < 0.0001* and is highly statistically significant. PE, Preeclampsia; MMRDEGs, Mitochondrial energy metabolism related differentially expressed genes; LASSO, Least Absolute Shrinkage and Selection Operator; Common MMRDEGs, Common Mitochondrial energy metabolism related differentially expressed genes.

Based on the Combined dataset, we examined the expression levels of the 16 MMRDEGs using Random Forest algorithm (RF) to evaluate the values in diagnosis of the 16 MMRDEGs (**Figure 5A**). IncNodePurity (Increase in NodePurity) indicates the enhancement in node purity. The higher the node purity, the less impurities it contains (that is, the smaller the Gini coefficient). We applied an IncNodePurity threshold of > 0.5 to filter the specific analysis outcomes. The findings (**Figure 5B**) revealed that 7 diagnostic markers were obtained by RF algorithm. They are: *OCRL*, *GAPDH*, *TPI1*, *LDHA*, *SOD1*, *HK2* and *PGK1*.

Logistic regression was performed utilizing the expression levels of 16 MMRDEGs in the Combined dataset, with the screening criterion of *p<* 0.05 (**Figure 5C**). The Logistic regression model included a total of 8 MMRDEGs (*ALDH16A1*, *ATG7*, *BTD*, *GAPDH*, *HK2*, *LDHA*, *OCRL* and *TPI1*), and the diagnostic model was developed by the expression relative quantities of the 8 genes in the combined dataset (the expression levels were evaluated by Least Absolute Shrinkage and Selection Operator (LASSO) analysis). And the findings of the LASSO analysis were illustrated via the LASSO regression model diagram (**Figure 5D**) and the LASSO variable trajectory plot (**Figure 5E**). The findings indicated that the diagnostic model comprised 6 MMRDEGs, which were: *ALDH16A1*, *ATG7*, *GAPDH*, *LDHA*, *OCRL* and *TPI1*.

Then we interposed the MMRDEGs from the RF model and the MMRDEGs from the Logistic-LASSO regression model (**Figure 5F**), and 4 Common MMRDEGs (*p < 0.05*) were obtained, which were *OCRL*, *GAPDH*, *TPI1* and *LDHA*.

Next, we examined the differential expression of the 4 Common MMRDEGs in the placental tissues of preeclamptic and normal mothers using RT-qPCR. The demographic characteristics of the PE patients are presented in **Supplementary Table 7**. The findings indicated that the mRNA expressions of the 4 common genes were notably elevated in the placental tissues of the PE cases relative to the Control group (*p < 0.05*, **Figure 5G**). These four Common MMRDEGs (*OCRL, GAPDH, TPI1, and LDHA*) were identified for the first time in a study of PE. This novel discovery offers fresh insights into the role of mitochondrial metabolism in preeclampsia and may establish a foundation for the development of future biomarkers and therapeutic targets.

And then, utilizing the expression level of the four Common MMRDEGs in Combined dataset and corresponding coefficients established by applying LASSO analysis, we obtained the MMRDEGs diagnostic model of 4 Common MMRDEGs.

$$\begin{array}{c} \text{Risk Score }=41.58006654+OCRL*-3.921473316+GAPDH*\\ -2.021501079+TPI1*-0.275314264+LDHA*-0.08998086 \end{array}$$

**Figure 6:**
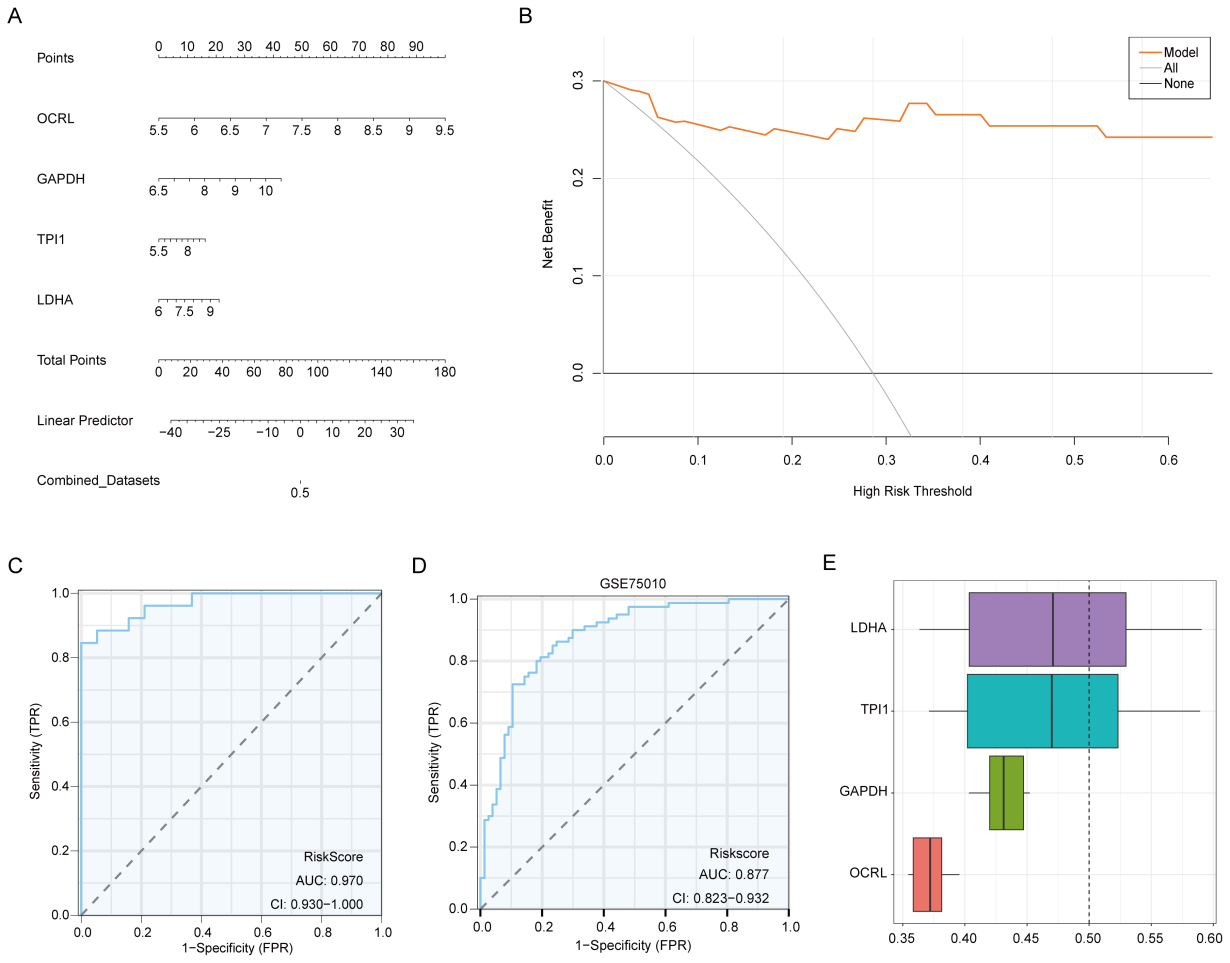
Validation of the MMRDEGs diagnostic model. **(A)** Nomogram of Common MMRDEGs in MMRDEGs Logistic regression model. **(B)** Decision curve in Logistic regression model of MMRDEGs. **(C)** ROC curve of MMRDEGs diagnostic model in Combined dataset. **(D)** ROC curve of MMRDEGs diagnostic model in GSE75010. **(E)** Functional similarity analysis results among Common MMRDEGs. ROC, receiver operating characteristic curve; AUC, Area Under the Curve, MMRDEGs, Mitochondrial energy metabolism related differentially expressed genes; Common MMRDEGs, Common Mitochondrial energy metabolism related differentially expressed genes; DCA, Decision Curve Analysis. The closer the AUC in the ROC curve is to 1, the better the diagnostic effect is. When AUC was between 0.5 and 0.7, the accuracy was low. When AUC was 0.7-0.9, it had a certain accuracy. AUC > 0.9 had high accuracy.

The diagnostic model for MMRDEGs included four Common MMRDEGs. We used combined logistic regression analysis to process the dataset’s expression levels to construct a logistic regression model for MMRDEGs. Additionally, we generated a nomogram depicting the impact of these four common MMRDEGs on the logistic regression model (**Figure 6A**). Our findings revealed that among all variables, *OCRL* exhibited notably superior effectiveness within the MMRDEGs logistic regression model.

The diagnostic model’s clinical value was appraised through decision curve analysis (DCA), and the findings were presented in **Figure 6B**. In the DCA graph, a model’s line consistently surpasses those of “All negative” and “All positive” within a specific range, greater net benefits can be obtained, indicating a stronger model performance. Our findings demonstrate that our constructed model exhibits considerable accuracy in diagnosing PE.

To further substantiate the value of the MMRDEGs diagnostic model, we drew ROC curves utilizing the Risk Scores of the diagnostic model of MMRDEGs and the information for grouping (PE/Control) of the Combined dataset and displayed the outcomes (**Figure 6C**). The MMRDEGs diagnostic model exhibited substantial precision in the diagnosis of the two groups (PE/Control) (AUC = 0.970, CI=0.930-1.000, **Figure 6C**).

We further validated the diagnostic performance of the MMRDEGs diagnostic model using the GSE75010 dataset. Specifically, we calculated the risk scores by applying the formula derived from the MMRDEGs diagnostic model and the gene expression profiles in GSE75010. Subsequently, we incorporated the grouping information to construct the ROC curve. The results indicated that the MMRDEGs diagnostic model exhibited satisfactory accuracy in distinguishing the PE and Control groups within the GSE75010 dataset (AUC = 0.877, CI 0.823-0.932, **Figure 6D**).

We also performed functional similarity analysis for four Common MMRDEGs and displayed them using a boxplot. We calculated the semantic similarity of sets of GO terms, GO terms, gene products and gene clusters through the R package GOSemSim. Similarity analysis was performed only on genes that were annotated to pathways in MF, BP, and CC. Finally, functional similarity analysis results between four Common MMRDEGs were obtained and visualized by Boxplot (**Figure 6E**). The findings indicated that LDHA exhibited the greatest functional similarity score in comparison to other Common MMRDEGs (the X-axis of D graph is the similarity score, with higher values indicating increased functional similarity to other genes).

### 3.6 GSEA and GSVA based on Combined dataset between the Low and the High-Risk cohorts

**Figure 7:**
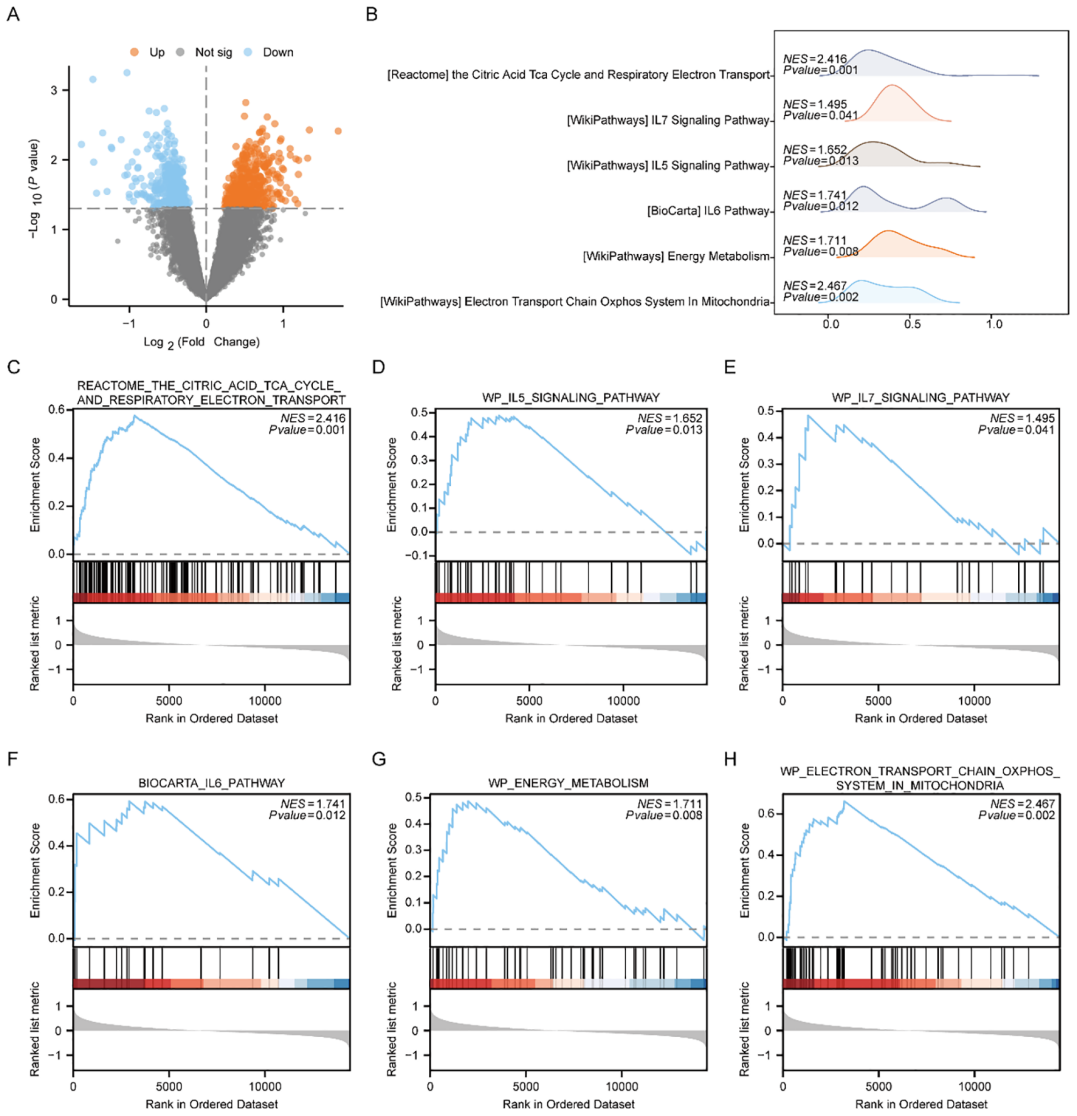
GSEA enrichment analysis between high and low risk-score groups of Combined dataset. **(A)** Volcano plot of gene difference analysis between High and Low Risk-score groups in Combined dataset. **(B)** Mountain plot display of six main biological characteristics of GSEA enrichment analysis results. C-H. Genes significantly enriched in the citric acid TCA cycle and respiratory electron transport between the High and Low Risk-score groups of Combined dataset **(C)**, IL7 signaling pathway **(D)**, IL5 signaling pathway **(E)**, IL6 pathway **(F)**, energy metabolism **(G)**, electron transport chain Oxphos system in mitochondria **(H)**. PE, Preeclampsia; GSEA, Gene Set Enrichment Analysis. The significant enrichment screening criterion for GSEA enrichment analysis was *p < 0.05*.

Initially, we categorized those disease samples from the Combined dataset into the Low-Risk Score group and the High-Risk Score group utilizing the median Risk-Score of the previous MMRDEGs diagnostic model and performed a differential analysis between the two groups utilizing the limma package (**Figure 7A**). Based on the results of the differential analysis, we conducted GSEA to explore the relationship among the MFs, the CCs, the BPs and the expression of all genes involved between the different groups (Low/High Risk-Score group) in the Combined dataset, using the threshold of p < 0.05 for enrichment selection. The findings demonstrated a significant enrichment of genes linked to the citric acid TCA cycle and respiratory electron transport (**Figure 7C**), IL5 signaling pathway (**Figure 7D**), IL7 signaling pathway (**Figure 7E**), IL6 signaling pathway (**Figure 7F**), energy metabolism (**Figure 7G**), electron transport chain Oxphos system in mitochondria (**Figure 7H**) as well as other pathways, indicating their association with High and Low Risk cohorts (**Supplementary Table 8**). Furthermore, the GSEA outcomes of genes among the High Risk-Score cohort and the Low Risk-Score cohort in the Combined dataset were presented by mountain plot (**Figure 7B**).

To investigate the disparities among the High Risk-Score cohort and the Low Risk-Score cohort in the Combined dataset, we subsequently conducted GSVA. From the pathways with a *p < 0.05*, we identified 10 pathways with the highest and lowest logFC for further analysis (refer to **Supplementary Table 9** for detailed information), respectively. The GSVA results of all genes revealed significant differences in 20 pathways among the High and Low Risk-Score cohorts in the Combined dataset. These pathways include defective *CSF2RB* causes *SMDP5, IPS LCP* with *H3K4ME3* and *H3K27ME3*, Korkola choriocarcinoma *DN*, *FGFR3B* ligand binding and activation, Aml methylation Cluster *7 DN*, Turashvili breast carcinoma Ductal vs Lobular *DN*, *FTO* obesity variant mechanism, miscellaneous substrates, *PEPI* pathway, *ES LCP* with *H3K4ME3* and *H3K27ME3* angiogenic targets of *VHL HIF2A* up regulation Biocarta Myosin pathway *OPN* targets Cluster 3 *Myc* targets *DN CTNNB1* pathway and proliferation mesothelioma survival up schavolt targets of *TP53* and *TP63 MAPK11* targets Pujana breast cancer with *BRCA1* mutated *DN* regulation of *PTEN* localization. According to the GSVA outcomes, we analyzed the differential expression of 20 pathways among the Low-Risk cohort and the High-Risk cohort in the Combined dataset, and the specific differential analysis findings (**Supplementary Figure 4A**) was showed as a heatmap by the R package. Furthermore, we employed the Mann-Whitney U test to examine the group distinction level of 20 pathways between diverse cohorts in the Combined dataset and displayed the findings by group comparison plot (**Supplementary Figure 4B**). The findings suggested that all the expressions of the 20 pathways were markedly different among the Low-Risk cohort and High-Risk cohort in the Combined dataset (*p*<0.05).

### 3.7 Analysis of differences in ssGSEA immune characteristics among the Low and High-Risk groups in the Combined dataset

We categorized PE samples in the Combined dataset into the Low Risk-Score and the High Risk-Score cohorts by the median Risk-Score of the MMRDEGs diagnostic model.

**Figure 8:**
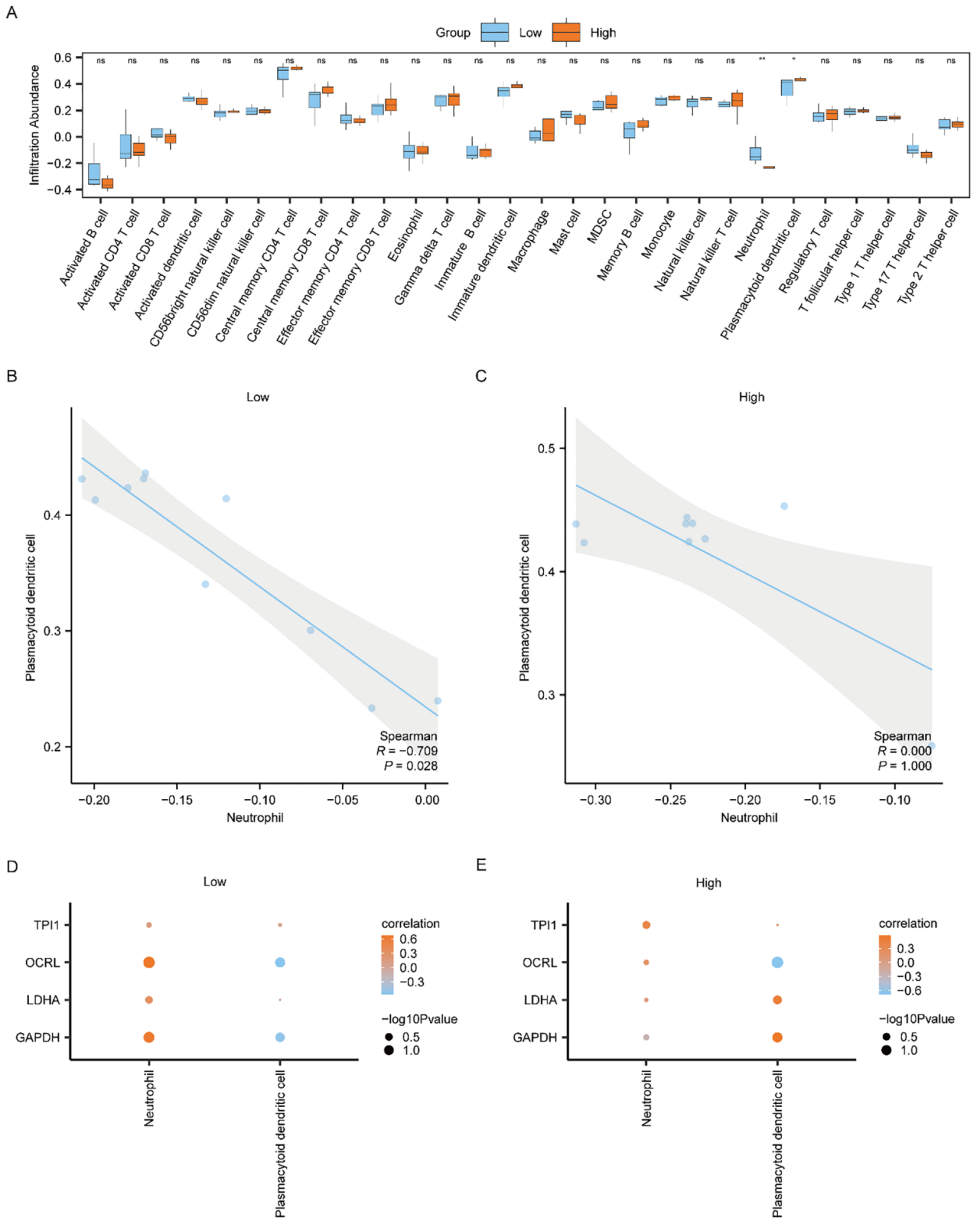
Differential analysis of ssGSEA immune characteristics between high and low risk-score groups in Combined dataset data. **(A)** The group comparison of ssGSEA immune infiltration analysis between the Low/High Risk-score groups of Combined dataset data. **(B, C)** Scatter plot of correlation between Neutrophil and Plasmacytoid dendritic cell of cell infiltration abundance in the Low Risk-score group **(B)** and High Risk-score group **(C)** of Combined dataset. **(D, E)** Dot plot of correlation between immune cells and Common MMRDEGs in the Low Risk-score group **(D)** and High Risk-score group **(E)** of Combined dataset. ssGSEA, single-sample gene-set enrichment Analysis; Common MMRDEGs, Common Mitochondrial energy metabolism related differentially expressed genes; PE, preeclampsia. The symbol ns is equivalent to *p ≥ 0.05* and not statistically significant; The symbol * is equivalent to *p < 0.05*, which is statistically significant; The symbol ** is equivalent to *p < 0.01*, which is highly statistically significant; The absolute value of the correlation coefficient in the scatter plot of correlation was more than 0.8, indicating a strong correlation. Moderate correlation was defined as an absolute value between 0.5 and 0.8. 0.3-0.5 is weak correlation; Values below 0.3 are considered weak or uncorrelated.

To study the difference of immune infiltration between the Low/High Risk-Score groups of the Combined dataset, we applied ssGSEA algorithm to computer the abundance of 28 immune cell infiltration in the two risk-score sample groups. And then, we used Mann-Whitney U test to analyze the differences of the two abundances of the Low and High Risk-Score groups, using group comparison plot to exhibit the results (**Figure 8A**). The findings suggested that there were two immune cells, namely Neutrophil and Plasmacytoid Dendritic cell, showing statistically differences in the abundance between the Low and High Risk-Score groups (*p < 0.05*).

We plotted the correlation scatter plots showing the relationship among Neutrophil and Plasmacytoid Dendritic cells in the Low-Risk cohort (**Figure 8B**) and the High-Risk cohort (**Figure 8C**) from the Combined dataset. The outcomes showed that, in the Low Risk-Score group, there was a marked inverse association among Neutrophil and Plasmacytoid Dendritic cells (**Figure 8B**, R = -0.709, *p* = 0.028). However, there was no association between the two immune cells in the High Risk-Score group (**Figure 8C**).

We used Spearman’s statistical algorithm to calculate the association between the infiltrating abundances of the Neutrophil, Plasmacytoid Dendritic cells in the Low and High Risk-Score cohorts, and the expression of the four Common MMRDEGs in the Combined dataset data group (**Figures 8D, E**). The findings suggested that Neutrophil was positively correlated with the four Common MMRDEGs in the Low Risk-Score cohort of the Combined dataset (**Figure 8D**), moreover Neutrophil and *OCRL* had the strongest association. In the High Risk-Score cohort of the Combined dataset data, Plasmacytoid Dendritic cells had the strongest correlation with *OCRL* (**Figure 8E**).

### 3.8 Cell-type Identification by Estimating Relative Subsets of RNA Transcripts (CIBERSORT) immunosignature comparative analysis among Low Risk-Score and High Risk-Score groups from the Combined dataset

The CIBERSORT method was utilized to estimate the abundance of 22 immune cell infiltrations in both the Low and High Score cohorts. A stacked bar chart was employed to graphically depict the distribution of immune cells across the dataset samples (**Supplementary Figure 5A**). There were 22 immune cells with non-zero infiltration abundances within the Combined dataset according to the results.

We used Spearman’s statistical algorithm to assess the relationships among the 22 immune cells (**Supplementary Figure 5B**), and the findings suggested that the number of positive and negative associations between the 22 immune cells was basically equal, among which Mast cells activated and B cells memory had the strongest correlation.

We subsequently computed the association among immune cells and the four Common MMRDEGs using Spearman’s statistical algorithm (**Supplementary Figure 5C**). The results showed that T cells CD4 memory activated, Dendritic cells resting, and T cells gamma delta were moderately positively correlated with the four Common MMRDEGs in the Combined dataset. Among all the associations examined, the most pronounced relationship was detected among naïve B cells and *GAPDH*.

### 3.9 PPI network and mRNA-RBP, mRNA-Drug and mRNA-TF interaction network were constructed

**Figure 9:**
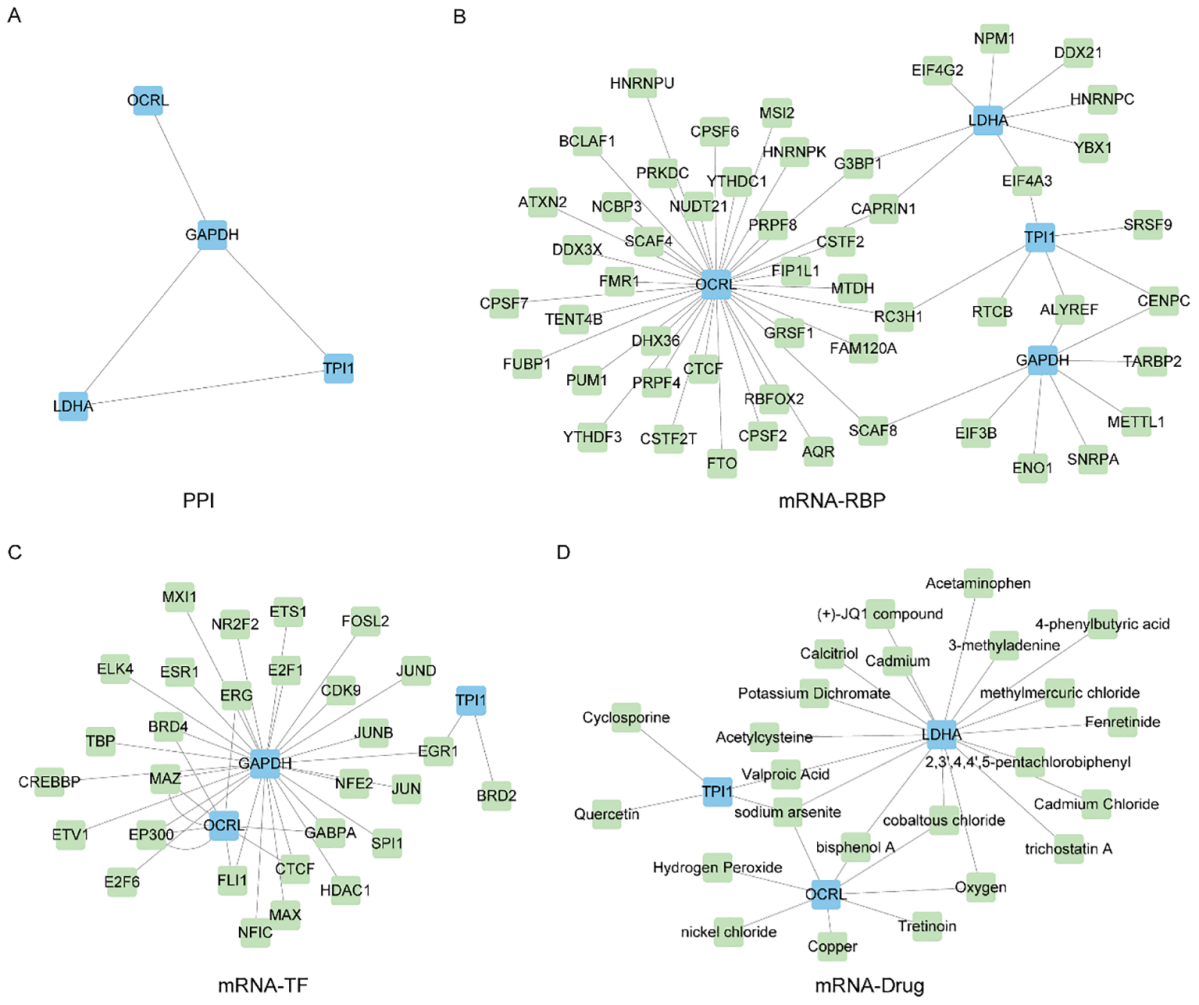
Construct PPI network and mRNA-RBP, mRNA-TF, mRNA-Drug interaction network. **(A)** Protein interaction network of Common MMRDEGs (PPI network). **(B)** mRNA-RBP network of Common MMRDEGs, blue quadrangle blocks are mRNA; Green quadrilateral blocks are RBP. **(C)** mRNA-TF network of Common MMRDEGs, and the blue quadrangle blocks in the mRNA-TF interaction network are mRNA; Green quadrangle-shaped blocks are TFs. **(D)** mRNA-Drug network of Common MMRDEGs, and the blue quadrangle blocks in the mRNA-Drug interaction network are mRNA; Green quadrangular blocks are drugs. PE, Preeclampsia; RBP, RNA binding protein; TFs, Transcription factors; Common MMRDEGs, Common Mitochondrial energy metabolism related differentially expressed genes.

Since these four Common MMRDEGs (*OCRL, GAPDH, TPI1, LDHA*) are the most potentially valuable biomarker genes identified during model construction, they are suspected to play a crucial role in related disease processes. Therefore, conducting an in-depth study on their interactions can aid in understanding their biological mechanisms and clinical applications. Therefore, we utilized the STRING database (PPI network, minimum required interaction score: low confidence (0.150)) to perform the PPI analysis of the 4 Common MMRDEGs (treated as hub genes) and visualized by Cytoscape software (**Figure 9A**).

And then, we utilized the ENCORI database to forecast RNA binding proteins (RBPs) that interacted with four Common MMRDEGs and subsequently visualized the mRNA-RBP interaction network by Cytoscape software (**Figure 9B**). The mRNA-RBP interaction network, in which green quadrilateral blocks presenting RBPs and the blue quadrilateral blocks presenting mRNAs, was composed of 4 Common MMRDEGs (*OCRL*, *GAPDH*, *TPI1* and *LDHA*) and 51 RBP molecules, which constituted 58 pairs of mRNA-RBP interaction relationships. The specific mRNA-RBP interaction relationships are depicted in **Supplementary Table 10**.

We utilized the CHIPBase database (version 3.0) and hTFtarget database to identify transcription factors (TFs) that bound to the four Common MMRDEGs. Then we screened by “Number of samples found (downstream)>0” and “Number of samples found (upstream) >0”, and finally got 3 Common MMRDEGs (*OCRL*, *GAPDH*, *TPI1*) and 39 pairs of interaction data of 29 TFs were graphically represented utilizing Cytoscape software (**Figure 9C**). In the mRNA-TF interaction network, those blue quadrilateral blocks represent mRNAs, and the green quadrilateral blocks are TFs. The detailed mRNA-TF interactions are depicted in the **Supplementary Table 11**.

We employed the Comparative Toxicogenomics Database (CTD) to identify small molecule compounds or potential drugs that interact with four commonly observed MMRDEGs. The selection criterion for mRNA-Drugs interaction pairs was set as “Reference Count” > 1. To render the mRNA-Drug interaction network (**Figure 9D**), we employed Cytoscape software. Within the mRNA-Drugs interaction network, the blue quadrilateral blocks signify mRNAs, while the green quadrilateral blocks denote drugs. Our analysis revealed that our mRNA-Drugs interaction network consisted of three common MMRDEGs (*OCRL, LDHA*, and *TPI1*) and twenty-four drug molecules, forming thirty mRNA-Drugs interaction associations. Detailed information regarding these specific interactions can be found in **Supplementary Table 12**.

**Figure 10:**
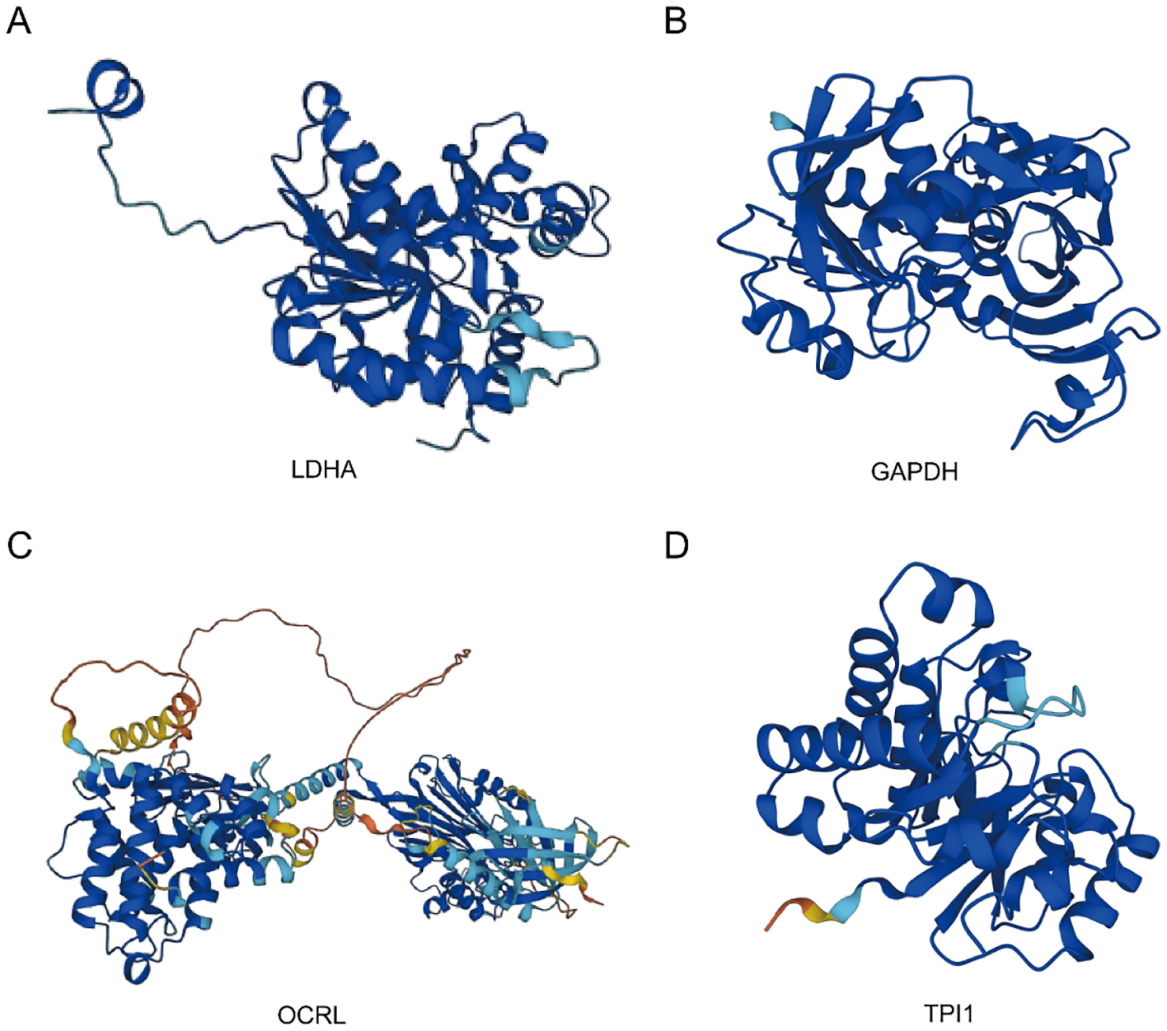
Protein structures of common MMRDEGs. The protein structures of LDHA **(A)**, GAPDH **(B)**, OCRL **(C)**, and TPI1 **(D)** are shown. The AlphaFold website produced a confidence score per residue (pLDDT) between 0 and 100. Some regions below 50 pLDDT may be isolated unstructured regions, and when pLDDT < 50 (red area), the model confidence is very low; When 50 < pLDDT < 70 (yellow area), the model confidence is low; When 70 < pLDDT < 90 (light blue area), the model confidence was normal. When 90 < pLDDT (blue area), the model confidence is very high. Common MMRDEGs, Common Mitochondrial energy metabolism related differentially expressed genes.

The AlphaFold Protein Structure Database (https://www.alphafold.ebi.ac.uk/) encompasses approximately 350,000 protein structure predictions generated by the AlphaFold AI system. This comprehensive database includes predictions for humans and 20 widely studied model organisms in biological research, such as E. coli, Drosophila, zebrafish, and mice. Remarkably, AlphaFold has successfully predicted the structures of 98.5% of human proteins within the human proteome. By combining AlphaFold’s structural prediction, we can more comprehensively construct and understand complex interaction networks, revealing the important roles of these genes in cellular metabolic regulation. This, in turn, provides a molecular basis for exploring the mechanisms of related diseases. To investigate the protein structures of four common MMRDEGs, we leveraged the resources provided by the AlphaFold website and presented our findings in **Figures 10A–D**.

## 4 Discussion

PE is a prevalent and severe complication of pregnancy, posing a substantial threat to both maternal and infant health. Its prognosis is intricately linked to maternal and infant outcomes. The primary clinical manifestations include hypertension, proteinuria, as well as liver and kidney impairment (59). Currently, the precise pathogenesis of PE remains incompletely elucidated. Study indicates that mitochondrial dysfunction is a pivotal factor in the development and progression of PE, with marked mitochondrial abnormalities being detected in PE patient (22, 60). The level of oxidative stress in patients with PE is significantly elevated. The excessive production of oxygen free radicals can induce damage to placental trophoblast cells (58, 61), while mitochondrial dysfunction further exacerbates oxidative stress, creating a vicious cycle that worsens the progression of PE (58). Mitochondria are the primary organelles responsible for cellular energy production. Impairment in mitochondrial function leads to a decrease in energy supply, which may contribute to elevated blood pressure, proteinuria, and multi-organ dysfunction among patients diagnosed with PE (62, 63). In addition, the immune response in patients with PE is markedly enhanced (8, 64). Another study suggests that immune system dysregulation may be closely associated with mitochondrial dysfunction (65), thus implying that immune dysregulation could contribute to the development of PE. Moreover, studies have further demonstrated that PE patients face a significantly elevated risk of developing hypertension and cardiovascular disease later in life (66, 67). PE can also result in FGRs, intrauterine distress, preterm delivery, and even intrauterine death due to its impact on maternal-fetal blood supply and oxygen delivery (68, 69). Therefore, the early diagnosis of PE is essential for enabling timely intervention and effectively reducing maternal and infant risks (70, 71). Currently, clinical screening primarily relies on the measurement of blood pressure and proteinuria (72), along with evaluations of edema, liver and kidney function (73, 74). However, these methods have limitations in terms of sensitivity and specificity.

Through integrative analysis of the GSE24129, GSE30186, and GSE54618 datasets, we identified 1,073 DEGs between PE cases and Control group. Subsequent comparative intersection analysis with MMRGs revealed 24 MMRDEGs. Notably, 16 of these MMRDEGs demonstrated significant differential expression patterns between PE cases and Control group. These DEGs may be associated with the development of PE, especially those MMRDEGs, which may affect cellular energy production and metabolic processes and play important roles in PE. Mitochondrial energy metabolism is a common metabolic pathway in tumor cells, and MMRDEGs may include key genes for hypoxia, oxidative stress and programmed cell death. Aberrant expression of these genes may lead to impaired chorionic trophoblast cell function, which in turn affects the development and prognosis of PE.

In this investigation, our findings revealed that *ATG7* expression was markedly decreased in PE cases. *ATG7* is an important component of early autophagy that encodes the E1 ubiquitin-activating enzyme, and its absence can lead to defective autophagy in the uterine vascular microenvironment, which in turn reduces uterine vascular permeability (75, 76). Decreased *ATG7* expression was found to inhibit primary cilia formation and trophoblast invasion, which in turn led to poor pregnancy outcomes (77). However, we must also acknowledge the dual role of autophagy in both physiological and pathological states, as excessive inhibition of autophagy may similarly have negative impacts on placental function. Alzubaidi et al. discovered that *ATG7* was elevated expressed in placental tissues of PE patients (78). This contradiction indicates that our current understanding of the relationship between *ATG7* and PE is potentially inadequate. Therefore, it is crucial to clarify the role of *ATG7* in various environments. Future research, particularly longitudinal studies, will be essential to elucidate the precise role of *ATG7* in the pathogenesis of PE. The main function of *SOD1* is to reduce free radical damage to cells through redox reactions. Studies showed that Oxidative stress inhibited SOD1 expression in placental tissue, which was significantly decreased in L-NAME-induced preeclamptic mice (79, 80) and it align closely with the conclusions drawn in our study. *FOXO1* is a member of the *FOXO* family and is intimately linked to cellular autophagy (81). It was confirmed that *FOXO1* was highly expressed in placental tissues of PE patients, which is consistent with our findings (78).

Functional correlation analysis was performed to explore the 16 MMRDEGs, we acquired a series of crucial insights regarding the PE pathogenesis. Firstly, GO analysis findings indicated that these 16 genes were primarily enriched in pyruvate metabolism, glycolysis, and ATP metabolism. Furthermore, KEGG analysis demonstrated that these genes are linked to processes such as glycolysis/glycolysis, HIF-1 signaling pathway, carbon metabolism, inositol phosphate metabolism, alanine, aspartate and glutamate metabolism. It has been established that placental mitochondrial dysfunction is prevalent in preeclampsia, while the inability to upregulate glycolysis is significantly correlated with increased disease severity (82). Pyruvate, a key product of glycolysis, plays an essential role in the production of reducing equivalents within mitochondria, ATP synthesis, and biosynthesis pathways such as glucose, fatty acids, and amino acids. Pyruvate metabolism is crucial for maintaining carbon homeostasis, and its dysregulation has been linked to various diseases, including diabetes, cancer, Embryogenesis, and cardiovascular disorders (83, 84). HK-2 exhibits phosphotransferase activity, alcohol-group receptor activity, and fructokinase activity. Relevant pathways include glycolysis and GDP-glucose biosynthesis II. Studies demonstrated that HK-2 is involved in glycolytic flux and mitochondrial activity during maladaptive inflammation in brain diseases. Additionally, HK-2 may exert therapeutic effects in osteoarthritis by modulating glucose metabolism (85–87). PGK-1, a glycolytic enzyme, is associated with glycolysis and gluconeogenesis pathways. Studies have shown that PGK-1 plays a significant role in neurodegenerative diseases (88, 89). Various studies have investigated the pivotal role of hypoxia-inducible factor-1 (HIF-1) in metabolic reprogramming across multiple pathways, including glycolysis, glycogen synthesis, lipid metabolism, the electron transport chain (ETC), the tricarboxylic acid (TCA) cycle, glutamine and serine metabolism, ROS production, as well as mitochondrial biogenesis and autophagy (90, 91). Abnormal expression of DNA and histone proteins represents a key characteristic of tumor cells. Their nucleotide metabolism and epigenetic regulation rely on the one-carbon metabolic pathway to preserve genomic stability and integrity (92). Given the further potential regulatory functions of mitochondria in abnormal energy metabolism, it offers a novel perspective for investigating the mechanism of preeclampsia. Finally, GSEA and GSVA analyses demonstrated a significant enrichment of genes from different (PE/Control) groups in the Combined dataset, specifically in the vascular smooth muscle contraction pathway, IL9 signaling pathway, Notch signaling pathway, IL2 signaling pathway, IL6/7 signaling pathway, cell surface interactions at the vascular wall. These gene clusters are critically involved in hypertension pathogenesis, immune regulation, inflammatory responses, and redox homeostasis maintenance through interconnected molecular pathways (93–96). These findings offer valuable insights and directions for further exploration of the pathogenesis of PE.

In the study, we constructed a diagnostic model containing four Common MMRDEGs (*OCRL, GAPDH, TPI1*, *LDHA)*, and verified that the model had high accuracy (AUC = 0.970) by ROC curve. Additionally, an external validation dataset was employed to assess the applicability of the model, and the results showed that the model achieved satisfactory accuracy for diagnosing PE. These four Common MMRDEGs not only showed significant differential expression, but also functional similarity among them. *OCRL* encodes an inositol polyphosphate 5-phosphatase that acts on phosphoinositide, which is a minor component of cell membranes but is a key regulator of intracellular transport (97, 98). *OCRL* catalyzes the production of the second messenger inositol triphosphate (IP3) and diacylglycerol (DAG) via phosphatidylinositol metabolism, thereby activating calcium release from intracellular stores. Deficiency in *OCRL1* results in mitochondrial calcium overload, ultimately causing mitochondrial dysfunction and apoptosis in T cells (99). Study shows that DAG mediates diabetic hyperglycemia and its associated complications via the DAG-PKC signaling pathway (100). In addition, research has demonstrated that the uterine artery endothelium exhibits an adaptive increase in Ca2+/IP3 exchange during pregnancy, however, a capacity that is notably diminished in preeclampsia (101). Recent studies have shown that *OCRL* plays an important role in cell metabolism, oxidative stress and inflammatory response, which provides new perspectives for understanding its specific effects in PE (99, 102, 103). Drugs that regulate the expression or function of *OCRL* may help restore the normal metabolic state of the placenta and reduce oxidative stress and inflammation, thereby improving the prognosis of PE.

As a glycolytic enzyme, the main function of *GAPDH* is to catalyze the conversion of glyceraldehyde-3-phosphate to 1,3-bisphosphoglycerate, concomitantly generating ATP. Therefore, *GAPDH* is a critical energy source for cellular metabolism (104). In addition, *GAPDH* has a variety of non-glycolytic functions. For instance, regulation of RNA export, DNA repair, autophagy and cell death (105). Dimethyl fumarate exerts its anti-inflammatory effects by inhibiting glycolysis in immune cells through inhibit the catalytic activity of *GAPDH (* 106). Further functional validation and mechanism research may provide new targets and help for the early diagnosis and treatment of PE. *TPI1* regulates the interconversion between glyceraldehyde-3-phosphate and dihydroxyacetone phosphate during glycolysis and gluconeogenesis, therefore, it is essential in the modulation of energy metabolism. *TPI1* can function as an inhibitor to modulate NK cytotoxicity via the SHP-1-ERK-STAT3 pathway (107). And the Erk signaling pathway has a direct impact on trophoblast proliferation (108). In addition, an increasing number of studies indicated that this gene influences glycolysis in target cells via different pathways, such as the METTL5/cMyc/TPI1 pathway, thereby affecting the onset and prognosis of various diseases, including lung cancer, liver cancer, and myopia (109–111). *LDHA* is widely present in the cytoplasm and can also be expressed in mitochondria and nucleus, which participate in and regulate cellular energy metabolism and have an important impact on cellular function (112). *LDHA* depletion leads to a reduction in ATP production, consequently diminishing PI3K-AKT-Foxo1 signaling and impairing the redox responses of effector T cells (113). Yang M et al. showed that glucose transporter 1 plays a critical role in glucose uptake and subsequent metabolic utilization. Knockdown of *GLUT1* reduced glucose uptake and suppressed lactate production by modulating the mRNA expression of *LDHA*, resulting in impairment of blastocyst implantation, trophoblast invasion, and placental development (114). Furthermore, we validated their expression in placental tissues using RT-qPCR assay. The before mentioned metabolic and immune disorders were found to be consistent with the impaired mitochondrial function, reduced ATP synthesis, and abnormal immune cell function observed in the placenta of patients with PE. These findings present a novel perspective on potential early diagnostic biomarkers for PE. The diagnostic model combined the expression levels of these genes and successfully differentiated between PE and Control group samples, suggesting their potential utility as diagnostic indicators for pregnancy-related hypertensive conditions.

GSEA and GSVA analyses revealed multiple pathways that exhibited marked differences among the Low and High-risk groups in the Combined dataset, encompassing various biological processes such as redox reactions, immune responses, and cell cycle regulation. Significantly enriched or altered genes in these pathways may have different impacts on Low and High-risk cohorts, leading to significant differences in immune status and cellular function between patients at different risk levels. This provides new insights for understanding risk assessment in PE patients and potential targets for future therapeutic strategies. Additionally, it confirmed the biological validity of the MMRDEGs correlation diagnosis model.

Existing research indicates that PE is a complex pregnancy-related disease involving multiple pathological mechanisms, including abnormal immune system responses. There is an increase in biomarkers indicating activation of the terminal complement pathway (115, 116). Deer et al. emphasized that immune cells such as regulatory T cells, macrophages, natural killer cells, and neutrophils are known to play major causal roles in the pathology of preeclampsia in their review (117). Aneman et al. further explored the distinct manifestations of the innate immune system in early and late stages of PE, positing that understanding immune cells holds the key to unveiling the pathogenesis of PE (118). In addition, Nieves et al. explored the impact of autoimmune diseases and infections on PE, highlighting that these factors can significantly exacerbate the condition (119). Lastly, Luo et al. uncovered immune interference at the maternal-fetal interface in PE via single-cell analysis and discussed HLA-F-mediated immune tolerance (120). Our study employed the ssGSEA and CIBERSORT algorithms to analyze immune cell infiltration characteristics between High-Risk and Low-Risk groups. Our study suggests that differential expression of neutrophils and plasmacytoid dendritic cells between these two groups, with neutrophils showing a positive correlation with four common MMRDEGs in the Low-Risk group. And among the 22 types of immune cells with non-zero infiltration abundance, Mast cells and B memory cells exhibited the strongest correlation.

Neutrophils constitute a critical component of the innate immune system. They are recruited to sites of infection or damaged tissues via a series of coordinated processes, including rolling, adhesion, spreading, intravascular crawling, transepithelial migration, and chemotaxis-driven tissue infiltration. These functions depend on cytoskeletal reorganization and energy metabolism. Studies indicated that neutrophils possess the ability to adapt to various metabolic pathways, such as metabolic pathways involving glucose, lipids, and amino acids, during inflammation or in response to different disease states (121, 122). Notably, mitochondria serve as crucial sites for the metabolic processing of these nutrients. Neutrophil extracellular traps (NETs), induced by oxidative stress, represent a critical immune defense mechanism against external bacterial infections (123). Moreover, NETs enhance mitochondrial stability through the TLR4/PGC1α pathway (122). Elevated neutrophil levels have been documented in the peripheral blood and subcutaneous fat micro vessels of patients with PE (124, 125). Furthermore, studies have demonstrated that the activity of neutrophils is influenced by the alteration in the plasma expression levels of MMP-1 and PAF in patients with PE (124, 126). One experimental study indicated that neutrophils cultured in placental conditioned medium derived from women with PE exhibited significantly greater adherence to endothelial cells compared to those cultured in placental conditioned medium from controls, suggesting that factors influencing neutrophil quantity and function may originate from placental sources (124). These studies were consistent with the results of our study.

Dendritic cells (DCs) are professional antigen-presenting cells, and plasmacytoid dendritic cells (pDCs) are one subset of DCs. pDCs can secrete substantial amounts of IFN-α and IFN-β, as well as IL-6, IL-8, IL-12, and tumor necrosis factors (TNFs), via the activation of the Toll-like receptor (TLR) 7/9-MyD88-IRF7 pathway (127). During pregnancy, the primary role of DCs is to present paternal/fetal antigens to regulatory T cells, thereby maintaining immune tolerance at the maternal-fetal interface (128). Studies have demonstrated that the levels of pDCs in the serum of PE patients are significantly decreased compared to those of normal patients (129). In addition, research has shown that DCs display diminished responsiveness to stimulation by various TLRs ligands in PE patients compared to those in healthy pregnancy (130). Moreover, the expression level of TLR3 at the maternal-fetal interface in PE is significantly elevated (131). The upregulated expression of TLR3 may function as a protective mechanism to counteract the impaired responsiveness of DCs to the stimulation by various TLR ligands. These findings suggest that DC-mediated inflammation is involved in local regulation at the maternal-fetal interface and may plays a crucial role in the pathogenesis and progression of PE. Our immune infiltration analysis demonstrated a significant inverse correlation between neutrophils and pDCs within the low-risk group. Conversely, no such significant correlation was detected in the high-risk group. These results suggest that there is complex immune regulation mediated by neutrophils and pDCs in PE patients, which may play a critical role in its progression.

Immunological alterations constitute a critical component of the etiology of PE, characterized by the presence of autoantibodies, including agonistic autoantibodies against the angiotensin II type 1 receptor (AT1) and so on (132). Salby et al. identified the proportion of the B cell is elevated in PE patients, because of a significantly diminished expression of programmed cell death protein 1 (PD-1) on CD27+CD24hiCD38hi regulatory B cells (133). Experimental investigations have confirmed that B2 cells activated by placental ischemia can induce hypertension, activate circulating NK cells, and promote the production of AT1 agonistic autoantibodies in normally pregnant rats (132). Mast cells are typically activated in response to pathogen invasion, tissue injury, or infection firstly and can release cytokines to regulate the local inflammatory immune reaction (134). Previous studies have shown that mast cell-derived exosomal miR-181a-5p regulates the viability, migration, and invasion of HTR-8/SVneo cells through the YY1/MMP-9 pathway (135). And relevant studies have indicated that the average histamine concentration and mast cell density are higher in PE patients (136). Our analysis of the immune infiltration in non-zero abundance immune cells showed that Mast cells activated and B cells memory had the strongest correlation. Further supporting of the observation was that Mast cells regulate B cell function through secreted cytokines in diseases such as allergic rhinitis and pulmonary hypertension (137, 138). In addition, antibodies generated by the B cell lineage and cytokines such as interleukin-10 (IL-10) can substantially modulate the function of mast cells. This modulation can, in turn, promote or restrict the development of regulatory B cells via multiple mechanisms (134). This finding unveils the connection between mitochondrial metabolism and immune cell function, presenting a novel research avenue for future immunotherapy and targeted interventions targeting PE, offering a fresh perspective for its early diagnosis and intervention.

Finally, as the four Common MMRDEGs are the most potentially valuable biomarkers screened by the model constructed and they may play key roles in the pathogenesis of PE, we constructed the PPI, mRNA-Drug, mRNA-RBP and mRNA-TF interaction networks with the four common genes. We identified 51 RBPs genes that could be therapeutic targets for PE by analyzing gene nodes in the network. Then, we utilized the CTD database to forecast potential therapeutic agents or small molecule compounds for PE treatment, identifying 24 drug molecules. Furthermore, we displayed the protein structures of four common MMRDEGs by leveraging the resources of AlphaFold. The results provided molecular basis for exploring the mechanism of PE. However, the potential mechanism and role required more investigation.

However, there are several important limitations to this study that should be considered when interpreting the results. Firstly, the relatively small sample size of the combined dataset (45 total: 19 preeclampsia cases and 26 controls) may limit the generalizability of transcriptomics and machine learning approaches. Therefore, we validated the mRNA-level expression differences of MMRDEGs using RT-qPCR and conducted the external validation of an independent dataset. Additionally, the significant difference in gestational weeks at delivery between the PE group and the Control group, while clinically relevant to PE management, could introduce confounding factors into gene expression analysis. Future studies should focus on large-scale, multi-center cohorts to enhance the robustness and reliability of the findings and their clinical applicability. Furthermore, protein-level validation of these biomarkers and functional investigations using cell lines and animal models are essential to confirm their roles in the pathogenesis of PE and assess their potential as therapeutic targets.

## 5 Conclusion

In this paper, we comprehensively explored the pathogenesis of preeclampsia, constructed a scoring model, analyzed the relationship between MMRDEGs and immune micro-infiltration, and predicted potential therapeutic targets and drug molecules for PE by GO, KEGG, GSEA, and GSVA. Nevertheless, the specific pathogenesis and molecular targets still need to be further verified.

## Data Availability

The raw data supporting the conclusions of this article will be made available by the authors, without undue reservation.

## References

[1] Wu Y Li M Ying H Gu Y Zhu Y Gu Y. Mitochondrial quality control alterations and placenta-related disorders. Front Physiol. (2024) 15:1344951. doi: 10.3389/fphys.2024.1344951, PMID: 38390447 PMC10883312

[2] Taglauer ES Fernandez-Gonzalez A Willis GR Reis M Yeung V Liu X. Antenatal mesenchymal stromal cell extracellular vesicle therapy prevents preeclamptic lung injury in mice. Am J Respir Cell Mol Biol. (2022) 66:86–95. doi: 10.1165/rcmb.2021-0307OC, PMID: 34614384 PMC8803363

[3] Cipolla MJ Biller J. Persistent brain injury after preeclampsia. Neurology. (2017) 88:1216–7. doi: 10.1212/WNL.0000000000003773, PMID: 28235812

[4] Duffy J Cairns AE Richards-Doran D van 't Hooft J Gale C Brown M. A core outcome set for pre-eclampsia research: an international consensus development study. BJOG: an Int J obstetrics gynaecology. (2020) 127:1516–26. doi: 10.1111/1471-0528.16319, PMID: 32416644

[5] Xu X Zhu M Zu Y Wang G Li X Yan J. Nox2 inhibition reduces trophoblast ferroptosis in preeclampsia via the STAT3/GPX4 pathway. Life Sci. (2024) 343:122555. doi: 10.1016/j.lfs.2024.122555, PMID: 38460811

[6] Murugesan S Hussey H Saravanakumar L Sinkey RG Sturdivant AB Powell MF. Extracellular vesicles from women with severe preeclampsia impair vascular endothelial function. Anesth analgesia. (2022) 134:713–23. doi: 10.1213/ANE.0000000000005812, PMID: 34871190

[7] Hobson SR Gurusinghe S Lim R Alers NO Miller SL Kingdom JC. Melatonin improves endothelial function *in vitro* and prolongs pregnancy in women with early-onset preeclampsia. J pineal Res. (2018) 65:e12508. doi: 10.1111/jpi.2018.65.issue-3, PMID: 29766570

[8] Horvat Mercnik M Schliefsteiner C Sanchez-Duffhues G Wadsack C. TGFβ signalling: a nexus between inflammation, placental health and preeclampsia throughout pregnancy. Hum Reprod Update. (2024) 30:442–471. doi: 10.1093/humupd/dmae007, PMID: 38519450 PMC11215164

[9] Overton E Tobes D Lee A. Preeclampsia diagnosis and management. Best Pract Res Clin anaesthesiology. (2022) 36:107–21. doi: 10.1016/j.bpa.2022.02.003, PMID: 35659948

[10] Phipps EA Thadhani R Benzing T Karumanchi SA. Pre-eclampsia: pathogenesis, novel diagnostics and therapies. Nat Rev Nephrology. (2019) 15:275–89. doi: 10.1038/s41581-019-0119-6, PMID: 30792480 PMC6472952

[11] Xiong D Yin Z Huang M Wang Y Hardy M Kalyanaraman B. Mitochondria-targeted atovaquone promotes anti-lung cancer immunity by reshaping tumor microenvironment and enhancing energy metabolism of anti-tumor immune cells. Cancer Commun (London England). (2024) 44:448–52. doi: 10.1002/cac2.12500, PMID: 37930151 PMC10958673

[12] Zou B Jia F Ji L Li X Dai R. Effects of mitochondria on postmortem meat quality: characteristic, isolation, energy metabolism, apoptosis and oxygen consumption. Crit Rev Food Sci Nutr. (2023) 64:11239–62. doi: 10.1080/10408398.2023.2235435, PMID: 37452658

[13] Su L Zhang J Gomez H Kellum JA Peng Z. Mitochondria ROS and mitophagy in acute kidney injury. Autophagy. (2023) 19:401–14. doi: 10.1080/15548627.2022.2084862, PMID: 35678504 PMC9851232

[14] Haridevamuthu B Murugan R Seenivasan B Meenatchi R Pachaiappan R Almutairi BO. Synthetic azo-dye, Tartrazine induces neurodevelopmental toxicity via mitochondria-mediated apoptosis in zebrafish embryos. J hazardous materials. (2024) 461:132524. doi: 10.1016/j.jhazmat.2023.132524, PMID: 37741213

[15] Yang L Yao C Su Z Fang Y Pandey NK Amador E. Combination of disulfiram and Copper-Cysteamine nanoparticles induces mitochondria damage and promotes apoptosis in endometrial cancer. Bioactive materials. (2024) 36:96–111. doi: 10.1016/j.bioactmat.2024.02.009, PMID: 38440322 PMC10911931

[16] Ticiani E Gingrich J Pu Y Vettathu M Davis J Martin D. Bisphenol S and epidermal growth factor receptor signaling in human placental cytotrophoblasts. Environ Health perspectives. (2021) 129:27005. doi: 10.1289/EHP7297, PMID: 33605785 PMC7894408

[17] Li J Quan X Zhang Y Yu T Lei S Huang Z. PPARγ Regulates triclosan induced placental dysfunction. Cells. (2021) 11:86. doi: 10.3390/cells11010086, PMID: 35011648 PMC8750171

[18] Huang P Song Y Yang Y Bai F Li N Liu D. Identification and verification of diagnostic biomarkers based on mitochondria-related genes related to immune microenvironment for preeclampsia using machine learning algorithms. Front Immunol. (2023) 14:1304165. doi: 10.3389/fimmu.2023.1304165, PMID: 38259465 PMC10800455

[19] Fisher JJ Vanderpeet CL Bartho LA McKeating DR Cuffe JSM Holland OJ. Mitochondrial dysfunction in placental trophoblast cells experiencing gestational diabetes mellitus. J Physiol. (2021) 599:1291–305. doi: 10.1113/tjp.v599.4, PMID: 33135816

[20] Juan-Reyes SS Gómez-Oliván LM Juan-Reyes NS Islas-Flores H Dublán-García O Orozco-Hernández JM. Women with preeclampsia exposed to air pollution during pregnancy: Relationship between oxidative stress and neonatal disease - Pilot study. Sci total environment. (2023) 871:161858. doi: 10.1016/j.scitotenv.2023.161858, PMID: 36716872

[21] Cipolla MJ Tremble SM DeLance N Johnson AC. Worsened stroke outcome in a model of preeclampsia is associated with poor collateral flow and oxidative stress. Stroke. (2023) 54:354–63. doi: 10.1161/STROKEAHA.122.041637, PMID: 36689585 PMC9888018

[22] Long J Huang Y Tang Z Shan Y Feng D Wang W. Mitochondria targeted antioxidant significantly alleviates preeclampsia caused by 11β-HSD2 dysfunction via OPA1 and mtDNA maintenance. Antioxidants (Basel Switzerland). (2022) 11:1505. doi: 10.3390/antiox11081505, PMID: 36009224 PMC9404992

[23] Pearce SF Rebelo-Guiomar P D'Souza AR Powell CA Haute LV Minczuk M. Regulation of mammalian mitochondrial gene expression: recent advances. Trends Biochem Sci. (2017) 42:625–39. doi: 10.1016/j.tibs.2017.02.003, PMID: 28285835 PMC5538620

[24] Ricci CA Reid DM Sun J Santillan DA Santillan MK Phillips NR. Maternal and fetal mitochondrial gene dysregulation in hypertensive disorders of pregnancy. Physiol Genomics. (2023) 55:275–85. doi: 10.1152/physiolgenomics.00005.2023, PMID: 37184228 PMC10292966

[25] Pandey D Yevale A Naha R Kuthethur R Chakrabarty S Satyamoorthy K. Mitochondrial DNA copy number variation - A potential biomarker for early onset preeclampsia. Pregnancy hypertension. (2021) 23:1–4. doi: 10.1016/j.preghy.2020.10.002, PMID: 33160129

[26] Deng YJ Ren EH Yuan WH Zhang GZ Wu ZL Xie QQ. GRB10 and E2F3 as diagnostic markers of osteoarthritis and their correlation with immune infiltration. Diagnostics (Basel). (2020) 10:171. doi: 10.3390/diagnostics10030171, PMID: 32235747 PMC7151213

[27] Dunk CE Bucher M Zhang J Hayder H Geraghty DE Lye SJ. Human leukocyte antigen HLA-C, HLA-G, HLA-F, and HLA-E placental profiles are altered in early severe preeclampsia and preterm birth with chorioamnionitis. Am J Obstetrics Gynecology. (2022) 227:641.e641–641.e613. doi: 10.1016/j.ajog.2022.07.021, PMID: 35863458

[28] Zhao Y Zhang X Du N Sun H Chen L Bao H. Immune checkpoint molecules on T cell subsets of pregnancies with preeclampsia and gestational diabetes mellitus. J Reprod Immunol. (2020) 142:103208. doi: 10.1016/j.jri.2020.103208, PMID: 33002799

[29] Roberts JM. Preeclampsia epidemiology(ies) and pathophysiology(ies). Best Pract Res Clin Obstetrics Gynaecology. (2024) 94:102480. doi: 10.1016/j.bpobgyn.2024.102480, PMID: 38490067

[30] Duckitt K Harrington D. Risk factors for pre-eclampsia at antenatal booking: systematic review of controlled studies. Bmj. (2005) 330:565. doi: 10.1136/bmj.38380.674340.E0, PMID: 15743856 PMC554027

[31] Nishizawa H Ota S Suzuki M Kato T Sekiya T Kurahashi H. Comparative gene expression profiling of placentas from patients with severe pre-eclampsia and unexplained fetal growth restriction. Reprod Biol Endocrinol. (2011) 9:107. doi: 10.1186/1477-7827-9-107, PMID: 21810232 PMC3199758

[32] Meng T Chen H Sun M Wang H Zhao G Wang X. Identification of differential gene expression profiles in placentas from preeclamptic pregnancies versus normal pregnancies by DNA microarrays. Omics: J Integr Biol. (2012) 16:301–11. doi: 10.1089/omi.2011.0066, PMID: 22702245 PMC3369279

[33] Jebbink JM Boot RG Keijser R Moerland PD Aten J Veenboer GJM. Increased glucocerebrosidase expression and activity in preeclamptic placenta. Placenta. (2015) 36:160–9. doi: 10.1016/j.placenta.2014.12.001, PMID: 25552189

[34] Gibbs I Leavey K Benton SJ Grynspan D Bainbridge SA Cox BJ. Placental transcriptional and histologic subtypes of normotensive fetal growth restriction are comparable to preeclampsia. Am J Obstetrics Gynecology. (2018) 220:110.e1–110.e21. doi: 10.1016/j.ajog.2018.10.003, PMID: 30312585

[35] Barrett T Troup DB Wilhite SE Ledoux P Rudnev D Evangelista C. NCBI GEO: mining tens of millions of expression profiles–database and tools update. Nucleic Acids Res. (2007) 35:D760–765. doi: 10.1093/nar/gkl887, PMID: 17099226 PMC1669752

[36] Davis S Meltzer PS. GEOquery: a bridge between the gene expression omnibus (GEO) and bioConductor. Bioinf (Oxford England). (2007) 23:1846–7. doi: 10.1093/bioinformatics/btm254, PMID: 17496320

[37] Stelzer G Rosen N Plaschkes I Zimmerman S Twik M Fishilevich S. The geneCards suite: from gene data mining to disease genome sequence analyses. Curr Protoc Bioinf. (2016) 54:1.30.31–31.30.33. doi: 10.1002/0471250953.2016.54.issue-1 27322403

[38] Ye Z Zhang H Kong F Lan J Yi S Jia W. Comprehensive analysis of alteration landscape and its clinical significance of mitochondrial energy metabolism pathway-related genes in lung cancers. Oxid Med Cell Longev. (2021) 2021:9259297. doi: 10.1155/2021/9259297, PMID: 34970420 PMC8713050

[39] Leek JT Johnson WE Parker HS Jaffe AE Storey JD. The sva package for removing batch effects and other unwanted variation in high-throughput experiments. Bioinformatics. (2012) 28:882–3. doi: 10.1093/bioinformatics/bts034, PMID: 22257669 PMC3307112

[40] Li S Gao K Yao D. Comprehensive Analysis of angiogenesis associated genes and tumor microenvironment infiltration characterization in cervical cancer. Heliyon. (2024) 10:e33277. doi: 10.1016/j.heliyon.2024.e33277, PMID: 39021997 PMC11252983

[41] Yu G. Gene ontology semantic similarity analysis using GOSemSim. Methods Mol Biol (Clifton NJ). (2020) 2117:207–15. doi: 10.1007/978-1-0716-0301-7_11, PMID: 31960380

[42] Kanehisa M Goto S. KEGG: kyoto encyclopedia of genes and genomes. Nucleic Acids Res. (2000) 28:27–30. doi: 10.1093/nar/28.1.27, PMID: 10592173 PMC102409

[43] Yu G Wang LG Han Y He QY. clusterProfiler: an R package for comparing biological themes among gene clusters. Omics: J Integr Biol. (2012) 16:284–7. doi: 10.1089/omi.2011.0118, PMID: 22455463 PMC3339379

[44] Subramanian A Tamayo P Mootha VK Mukherjee S Ebert BL Gillette MA. Gene set enrichment analysis: a knowledge-based approach for interpreting genome-wide expression profiles. Proc Natl Acad Sci United States America. (2005) 102:15545–50. doi: 10.1073/pnas.0506580102, PMID: 16199517 PMC1239896

[45] Liberzon A Subramanian A Pinchback R Thorvaldsdóttir H Tamayo P Mesirov JP. Molecular signatures database (MSigDB) 3. 0 Bioinf. (2011) 27:1739–40. doi: 10.1093/bioinformatics/btr260, PMID: 21546393 PMC3106198

[46] Hänzelmann S Castelo R Guinney J. GSVA: gene set variation analysis for microarray and RNA-seq data. BMC Bioinf. (2013) 14:7. doi: 10.1186/1471-2105-14-7, PMID: 23323831 PMC3618321

[47] Liu Y Zhao H. Variable importance-weighted random forests. Quantitative Biol (Beijing China). (2017) 5:338–51. doi: 10.1007/s40484-017-0121-6 PMC6051549 30034909

[48] Engebretsen S Bohlin J. Statistical predictions with glmnet. Clin epigenetics. (2019) 11:123. doi: 10.1186/s13148-019-0730-1, PMID: 31443682 PMC6708235

[49] Park SY. Nomogram: An analogue tool to deliver digital knowledge. J Thorac Cardiovasc surgery. (2018) 155:1793. doi: 10.1016/j.jtcvs.2017.12.107, PMID: 29370910

[50] Tataranni T Piccoli C. Dichloroacetate (DCA) and cancer: an overview towards clinical applications. Oxid Med Cell longevity. (2019) 2019:8201079. doi: 10.1155/2019/8201079, PMID: 31827705 PMC6885244

[51] Charoentong P Finotello F Angelova M Mayer C Efremova M Rieder D. Pan-cancer immunogenomic analyses reveal genotype-immunophenotype relationships and predictors of response to checkpoint blockade. Cell Rep. (2017) 18:248–62. doi: 10.1016/j.celrep.2016.12.019, PMID: 28052254

[52] Barbie DA Tamayo P Boehm JS Kim SY Moody SE Dunn IF. Systematic RNA interference reveals that oncogenic KRAS-driven cancers require TBK1. Nature. (2009) 462:108–12. doi: 10.1038/nature08460, PMID: 19847166 PMC2783335

[53] Chen B Khodadoust MS Liu CL Newman AM Alizadeh AA. Profiling tumor infiltrating immune cells with CIBERSORT. Methods Mol Biol (Clifton NJ). (2018) 1711:243–59. doi: 10.1007/978-1-4939-7493-1_12, PMID: 29344893 PMC5895181

[54] von Mering C Huynen M Jaeggi D Schmidt S Bork P Snel B. STRING: a database of predicted functional associations between proteins. Nucleic Acids Res. (2003) 31:258–61. doi: 10.1093/nar/gkg034, PMID: 12519996 PMC165481

[55] Li JH Liu S Zhou H Qu LH Yang JH. starBase v2.0: decoding miRNA-ceRNA, miRNA-ncRNA and protein-RNA interaction networks from large-scale CLIP-Seq data. Nucleic Acids Res. (2014) 42:D92–97. doi: 10.1093/nar/gkt1248, PMID: 24297251 PMC3964941

[56] Zhang Q Liu W Zhang HM Xie JY Miao YR Xia M. hTFtarget: A comprehensive database for regulations of human transcription factors and their targets. Genomics Proteomics Bioinf. (2020) 18:120–8. doi: 10.1016/j.gpb.2019.09.006, PMID: 32858223 PMC7647694

[57] Torbergsen T øian P Mathiesen E Borud O. Pre-eclampsia-A mitochondrial disease? Acta Obstetricia Gynecologica Scandinavica. (1989) 68:145–8. doi: 10.3109/00016348909009902, PMID: 2589041

[58] Marín R Chiarello DI Abad C Rojas D Toledo F Sobrevia L. Oxidative stress and mitochondrial dysfunction in early-onset and late-onset preeclampsia. Biochim Biophys Acta Mol Basis Dis. (2020) 1866:165961. doi: 10.1016/j.bbadis.2020.165961, PMID: 32916282

[59] Dimitriadis E Rolnik DL Zhou W Estrada-Gutierrez J Koga K Francisco RPV. Pre-eclampsia. Nat Rev Dis primers. (2023) 9:8. doi: 10.1038/s41572-023-00417-6, PMID: 36797292

[60] Yung HW Colleoni F Dommett E Cindrova-Davies T Kingdom J Murray AJ. Noncanonical mitochondrial unfolded protein response impairs placental oxidative phosphorylation in early-onset preeclampsia. Proc Natl Acad Sci United States America. (2019) 116:18109–18. doi: 10.1073/pnas.1907548116, PMID: 31439814 PMC6731647

[61] Chiarello DI Abad C Rojas D Toledo F Vázquez CM Mate A. Oxidative stress: Normal pregnancy versus preeclampsia. Biochim Biophys Acta Mol Basis Dis. (2020) 1866:165354. doi: 10.1016/j.bbadis.2018.12.005, PMID: 30590104

[62] Hu XQ Zhang L. Hypoxia and the integrated stress response promote pulmonary hypertension and preeclampsia: Implications in drug development. Drug Discov Today. (2021) 26:2754–73. doi: 10.1016/j.drudis.2021.07.011, PMID: 34302972 PMC8612951

[63] Zhao Y Zhao H Xu H An P Ma B Lu H. Perfluorooctane sulfonate exposure induces preeclampsia-like syndromes by damaging trophoblast mitochondria in pregnant mice. Ecotoxicology Environ safety. (2022) 247:114256. doi: 10.1016/j.ecoenv.2022.114256, PMID: 36327784

[64] Li Y Sang Y Chang Y Xu C Lin Y Zhang Y. A galectin-9-driven CD11c(high) decidual macrophage subset suppresses uterine vascular remodeling in preeclampsia. Circulation. (2024) 149:1670–88. doi: 10.1161, PMID: 38314577 10.1161/CIRCULATIONAHA.123.064391

[65] Iborra-Pernichi M Ruiz García J Velasco de la Esperanza M Estrada BS Bovolenta ER Cifuentes C. Defective mitochondria remodelling in B cells leads to an aged immune response. Nat Commun. (2024) 15:2569. doi: 10.1038/s41467-024-46763-1, PMID: 38519473 PMC10960012

[66] Olivera S Graham D. Modelling pre-eclampsia and its cardiovascular effects. Nat Rev Cardiol. (2024) 21:281. doi: 10.1038/s41569-024-01006-0, PMID: 38480793

[67] Yang C Baker PN Granger JP Davidge ST Tong C. Long-term impacts of preeclampsia on the cardiovascular system of mother and offspring. Hypertension. (2023) 80:1821–33. doi: 10.1161/HYPERTENSIONAHA.123.21061, PMID: 37377011

[68] Stubert J Hinz B Berger R. The role of acetylsalicylic acid in the prevention of pre-eclampsia, fetal growth restriction, and preterm birth. Deutsches Arzteblatt Int. (2023) 120:617–26. doi: 10.3238/arztebl.m2023.0133, PMID: 37378599 PMC10568740

[69] Cluver CA Bergman L Bergkvist J Imberg H Geerts L Hall DR. Impact of fetal growth restriction on pregnancy outcome in women undergoing expectant management for preterm pre-eclampsia. Ultrasound obstetrics gynecology: Off J Int Soc Ultrasound Obstetrics Gynecology. (2023) 62:660–7. doi: 10.1002/uog.26282, PMID: 37289938 PMC10947051

[70] Benzing T. Hypertension: Testing for pre-eclampsia: paving the way to early diagnosis. Nat Rev Nephrology. (2016) 12:200–2. doi: 10.1038/nrneph.2016.21, PMID: 26923207

[71] Chaudhary RK Madaboosi N Satija J Nandagopal B Srinivasan R Sai VVR. Polymeric optical fiber biosensor with PAMAM dendrimer-based surface modification and PlGF detection for pre-eclampsia diagnosis. Biosensors bioelectronics. (2024) 257:116312. doi: 10.1016/j.bios.2024.116312, PMID: 38657380

[72] Wright A von Dadelszen P Magee LA Syngelaki A Akolekar R Wright D. Effect of race on the measurement of angiogenic factors for prediction and diagnosis of pre-eclampsia. BJOG: an Int J obstetrics gynaecology. (2023) 130:78–87. doi: 10.1111/1471-0528.17296, PMID: 36168103

[73] Andronikidi PE Orovou E Mavrigiannaki E Athanasiadou V Tzitiridou-Chatzopoulou M Iatrakis G. Placental and renal pathways underlying pre-eclampsia. Int J Mol Sci. (2024) 25:2741. doi: 10.3390/ijms25052741, PMID: 38473987 PMC10931599

[74] Ahmadian E Rahbar Saadat Y Hosseiniyan Khatibi SM Nariman-Saleh-Fam Z Bastami M Zununi Vahed F. Pre-Eclampsia: Microbiota possibly playing a role. Pharmacol Res. (2020) 155:104692. doi: 10.1016/j.phrs.2020.104692, PMID: 32070720

[75] Lee B Shin H Oh JE Park J Yang SC Jun JH. An autophagic deficit in the uterine vessel microenvironment provokes hyperpermeability through deregulated VEGFA, NOS1, and CTNNB1. Autophagy. (2021) 17:1649–66. doi: 10.1080/15548627.2020.1778292, PMID: 32579471 PMC8354601

[76] Nakashima A Cheng SB Ikawa M Yoshimori T Huber J Menon R. Evidence for lysosomal biogenesis proteome defect and impaired autophagy in preeclampsia. Autophagy. (2020) 16:1771–85. doi: 10.1080/15548627.2019.1707494, PMID: 31856641 PMC8386603

[77] Lin RC Chao YY Su MT Tsai HL Tsai PY Wang CY. Upregulation of miR-20b-5p inhibits trophoblast invasion by blocking autophagy in recurrent miscarriage. Cell signalling. (2024) 113:110934. doi: 10.1016/j.cellsig.2023.110934, PMID: 37871665

[78] Alzubaidi KRK Mahdavi M Dolati S Yousefi M. Observation of increased levels of autophagy-related genes and proteins in women with preeclampsia: a clinical study. Mol Biol Rep. (2023) 50:4831–40. doi: 10.1007/s11033-023-08385-6, PMID: 37039997

[79] Ye L Huang Y Liu X Zhang X Cao Y Kong X. Apelin/APJ system protects placental trophoblasts from hypoxia-induced oxidative stress through activating PI3K/Akt signaling pathway in preeclampsia. Free Radical Biol Med. (2023) 208:759–70. doi: 10.1016/j.freeradbiomed.2023.09.030, PMID: 37774802

[80] Martinez-Fierro ML Garza-Veloz I Castañeda-Lopez ME Wasike D la Rosa CCD Rodriguez-Sanchez IP. Evaluation of the effect of the fibroblast growth factor type 2 (FGF-2) administration on placental gene expression in a murine model of preeclampsia induced by L-NAME. Int J Mol Sci. (2022) 23:10129. doi: 10.3390/ijms231710129, PMID: 36077527 PMC9456139

[81] Santos BF Grenho I Martel PJ Ferreira BI Link W. FOXO family isoforms. Cell Death disease. (2023) 14:702. doi: 10.1038/s41419-023-06177-1, PMID: 37891184 PMC10611805

[82] Aye ILMH Aiken CE Charnock-Jones DS Smith GCS. Placental energy metabolism in health and disease&x2014;significance of development and implications for preeclampsia. Am J Obstetrics Gynecology. (2022) 226:S928–44. doi: 10.1016/j.ajog.2020.11.005, PMID: 33189710

[83] Yiew NKH Finck BN. The mitochondrial pyruvate carrier at the crossroads of intermediary metabolism. Am J Physiol Endocrinol Metab. (2022) 323:E33–e52. doi: 10.1152/ajpendo.00074.2022, PMID: 35635330 PMC9273276

[84] Prochownik EV Wang H. The metabolic fates of pyruvate in normal and neoplastic cells. Cells. (2021) 10:762. doi: 10.3390/cells10040762, PMID: 33808495 PMC8066905

[85] Hu Y Cao K Wang F Wu W Mai W Qiu L. Dual roles of hexokinase 2 in shaping microglial function by gating glycolytic flux and mitochondrial activity. Nat Metab. (2022) 4:1756–74. doi: 10.1038/s42255-022-00707-5, PMID: 36536134

[86] Fang J Luo S Lu Z. HK2: Gatekeeping microglial activity by tuning glucose metabolism and mitochondrial functions. Mol Cell. (2023) 83:829–31. doi: 10.1016/j.molcel.2023.02.022, PMID: 36931254

[87] Bao C Zhu S Song K He C. HK2: a potential regulator of osteoarthritis via glycolytic and non-glycolytic pathways. Cell Commun Signal. (2022) 20:132. doi: 10.1186/s12964-022-00943-y, PMID: 36042519 PMC9426234

[88] Siddique AHH Kale PP. Importance of glucose and its metabolism in neurodegenerative disorder, as well as the combination of multiple therapeutic strategies targeting α-synuclein and neuroprotection in the treatment of Parkinson's disease. Rev Neurologique. (2024) 180:736–53. doi: 10.1016/j.neurol.2023.08.011, PMID: 38040547

[89] Duncan L Shay C Teng Y. PGK1: an essential player in modulating tumor metabolism. Methods Mol Biol (Clifton NJ). (2022) 2343:57–70. doi: 10.1007/978-1-0716-1558-4_4, PMID: 34473315

[90] Infantino V Santarsiero A Convertini P Todisco S Iacobazzi V. Cancer cell metabolism in hypoxia: role of HIF-1 as key regulator and therapeutic target. Int J Mol Sci. (2021) 22:5703. doi: 10.3390/ijms22115703, PMID: 34071836 PMC8199012

[91] Zhao M Wang S Zuo A Zhang J Wen W Jiang W. HIF-1α/JMJD1A signaling regulates inflammation and oxidative stress following hyperglycemia and hypoxia-induced vascular cell injury. Cell Mol Biol Lett. (2021) 26:40. doi: 10.1186/s11658-021-00283-8, PMID: 34479471 PMC8414688

[92] Islam A Shaukat Z Hussain R Gregory SL. One-carbon and polyamine metabolism as cancer therapy targets. Biomolecules. (2022) 12:1902. doi: 10.3390/biom12121902, PMID: 36551330 PMC9775183

[93] Le Floc’h A Nagashima K Birchard D Scott G Ben L-H Ajithdoss D. Blocking common γ chain cytokine signaling ameliorates T cell–mediated pathogenesis in disease models. Sci Trans Med. (2023) 15:eabo0205. doi: 10.1126/scitranslmed, PMID: 36630481

[94] Touyz RM Alves-Lopes R Rios FJ Camargo LL Anagnostopoulou A Arner A. Vascular smooth muscle contraction in hypertension. Cardiovasc Res. (2018) 114:529–39. doi: 10.1093/cvr/cvy023, PMID: 29394331 PMC5852517

[95] Xiao L Ma X Ye L Su P Xiong W Bi E. IL-9/STAT3/fatty acid oxidation-mediated lipid peroxidation contributes to Tc9 cell longevity and enhanced antitumor activity. J Clin Invest. (2022) 132:e153247. doi: 10.1172/JCI153247, PMID: 35192544 PMC8970676

[96] Yang S Li F Lu S Ren L Bian S Liu M. Ginseng root extract attenuates inflammation by inhibiting the MAPK/NF-κB signaling pathway and activating autophagy and p62-Nrf2-Keap1 signaling *in vitro* and *in vivo* . J Ethnopharmacology. (2022) 283:114739. doi: 10.1016/j.jep.2021.114739, PMID: 34648903

[97] Erdmann KS Mao Y McCrea HJ Zoncu R Lee S Paradise S. A role of the Lowe syndrome protein OCRL in early steps of the endocytic pathway. Dev Cell. (2007) 13:377–90. doi: 10.1016/j.devcel.2007.08.004, PMID: 17765681 PMC2025683

[98] Hagemann N Hou X Goody RS Itzen A Erdmann KS. Crystal structure of the Rab binding domain of OCRL1 in complex with Rab8 and functional implications of the OCRL1/Rab8 module for Lowe syndrome. Small Gtpases. (2012) 3:107–10. doi: 10.4161/sgtp.19380, PMID: 22790198

[99] Chen H Lu C Tan Y Weber-Boyvat M Zheng J Xu M. Oculocerebrorenal syndrome of Lowe (OCRL) controls leukemic T-cell survival by preventing excessive PI(4,5)P(2) hydrolysis in the plasma membrane. J Biol Chem. (2023) 299:104812. doi: 10.1016/j.jbc.2023.104812, PMID: 37172724 PMC10279916

[100] Liu Y Wang X Nawaz A Kong Z Hong Y Wang C. Wogonin ameliorates lipotoxicity-induced apoptosis of cultured vascular smooth muscle cells via interfering with DAG-PKC pathway. Acta Pharmacologica Sinica. (2011) 32:1475–82. doi: 10.1038/aps.2011.120, PMID: 21986573 PMC4010212

[101] Ampey AC Dahn RL Grummer MA Bird IM. Differential control of uterine artery endothelial monolayer integrity by TNF and VEGF is achieved through multiple mechanisms operating inside and outside the cell - Relevance to preeclampsia. Mol Cell Endocrinol. (2021) 534:111368. doi: 10.1016/j.mce.2021.111368, PMID: 34153378 PMC8344923

[102] Li J Jiang L Kai H Zhou Y Cao J Tang W. Identifying preeclampsia-associated key module and hub genes via weighted gene co-expression network analysis. Sci Rep. (2025) 15:1364. doi: 10.1038/s41598-025-85599-7, PMID: 39779839 PMC11711461

[103] Lei C Chen W Wang Y Zhao B Liu P Kong Z. Prognostic prediction model for glioblastoma: A metabolic gene signature and independent external validation. J Cancer. (2021) 12:3796–808. doi: 10.7150/jca.53827, PMID: 34093788 PMC8176239

[104] Liu S Sun Y Jiang M Li Y Tian Y Xue W. Glyceraldehyde-3-phosphate dehydrogenase promotes liver tumorigenesis by modulating phosphoglycerate dehydrogenase. Hepatol (Baltimore Md). (2017) 66:631–45. doi: 10.1002/hep.29202, PMID: 28387968

[105] Sirover MA. The role of posttranslational modification in moonlighting glyceraldehyde-3-phosphate dehydrogenase structure and function. Amino Acids. (2021) 53:507–15. doi: 10.1007/s00726-021-02959-z, PMID: 33651246

[106] Kornberg MD Bhargava P Kim PM Putluri V Snowman AM Putluri N. Dimethyl fumarate targets GAPDH and aerobic glycolysis to modulate immunity. Science. (2018) 360:449–53. doi: 10.1126/science.aan4665, PMID: 29599194 PMC5924419

[107] Teng R Wang Y Lv N Zhang D Williamson RA Lei L. Hypoxia impairs NK cell cytotoxicity through SHP-1-mediated attenuation of STAT3 and ERK signaling pathways. J Immunol Res. (2020) 2020:4598476. doi: 10.1155/2020/4598476, PMID: 33123602 PMC7584946

[108] Pang H Lei D Huang J Guo Y Fan C. Elevated PGT promotes proliferation and inhibits cell apoptosis in preeclampsia by Erk signaling pathway. Mol Cell Probes. (2023) 67:101896. doi: 10.1016/j.mcp.2023.101896, PMID: 36731680

[109] Lin X Lei Y Pan M Hu C Xie B Wu W. Augmentation of scleral glycolysis promotes myopia through histone lactylation. Cell Metab. (2024) 36:511–525.e517. doi: 10.1016/j.cmet.2023.12.023, PMID: 38232735

[110] Liu P Sun SJ Ai YJ Feng X Zheng YM Gao Y. Elevated nuclear localization of glycolytic enzyme TPI1 promotes lung adenocarcinoma and enhances chemoresistance. Cell Death Dis. (2022) 13:205. doi: 10.1038/s41419-022-04655-6, PMID: 35246510 PMC8897412

[111] Xia P Zhang H Lu H Xu K Jiang X Jiang Y. METTL5 stabilizes c-Myc by facilitating USP5 translation to reprogram glucose metabolism and promote hepatocellular carcinoma progression. Cancer Commun (Lond). (2023) 43:338–64. doi: 10.1002/cac2.12403, PMID: 36602428 PMC10009668

[112] Dai M Wang L Yang J Chen J Dou X Chen R. LDHA as a regulator of T cell fate and its mechanisms in disease. Biomedicine pharmacotherapy = Biomedecine pharmacotherapie. (2023) 158:114164. doi: 10.1016/j.biopha.2022.114164, PMID: 36916398

[113] Xu K Yin N Peng M Stamatiades EG Shyu A Li P. Glycolysis fuels phosphoinositide 3-kinase signaling to bolster T cell immunity. Science. (2021) 371:405–10. doi: 10.1126/science.abb2683, PMID: 33479154 PMC8380312

[114] Yang M Li H Rong M Zhang H Hou L Zhang C. Dysregulated GLUT1 may be involved in the pathogenesis of preeclampsia by impairing decidualization. Mol Cell Endocrinology. (2022) 540:111509. doi: 10.1016/j.mce.2021.111509, PMID: 34801669

[115] Burwick RM Feinberg BB. Complement activation and regulation in preeclampsia and hemolysis, elevated liver enzymes, and low platelet count syndrome. Am J obstetrics gynecology. (2022) 226:S1059–s1070. doi: 10.1016/j.ajog.2020.09.038, PMID: 32986992

[116] Ho SJ Chaput D Sinkey RG Garces AH New EP Okuka M. Proteomic studies of VEGFR2 in human placentas reveal protein associations with preeclampsia, diabetes, gravidity, and labor. Cell communication signaling: CCS. (2024) 22:221. doi: 10.1186/s12964-024-01567-0, PMID: 38594674 PMC11003095

[117] Deer E Herrock O Campbell N Cornelius D Fitzgerald S Amaral LM. The role of immune cells and mediators in preeclampsia. Nat Rev Nephrol. (2023) 19:257–70. doi: 10.1038/s41581-022-00670-0, PMID: 36635411 PMC10038936

[118] Aneman I Pienaar D Suvakov S Simic TP Garovic VD McClements L. Mechanisms of key innate immune cells in early- and late-onset preeclampsia. Front Immunol. (2020) 11:1864. doi: 10.3389/fimmu.2020.01864, PMID: 33013837 PMC7462000

[119] Nieves C Victoria da Costa Ghignatti P Aji N Bertagnolli M. Immune cells and infectious diseases in preeclampsia susceptibility. Can J Cardiol. (2024) 40:2340–55. doi: 10.1016/j.cjca.2024.09.012, PMID: 39304126

[120] Luo F Liu F Guo Y Xu W Li Y Yi J. Single-cell profiling reveals immune disturbances landscape and HLA-F-mediated immune tolerance at the maternal-fetal interface in preeclampsia. Front Immunol. (2023) 14:1234577. doi: 10.3389/fimmu.2023.1234577, PMID: 37854606 PMC10579943

[121] Kumar S Dikshit M. Metabolic insight of neutrophils in health and disease. Front Immunol. (2019) 10:2099. doi: 10.3389/fimmu.2019.02099, PMID: 31616403 PMC6764236

[122] Yazdani HO Roy E Comerci AJ van der Windt DJ Zhang H Huang H. Neutrophil extracellular traps drive mitochondrial homeostasis in tumors to augment growth. Cancer Res. (2019) 79:5626–39. doi: 10.1158/0008-5472.CAN-19-0800, PMID: 31519688 PMC6825588

[123] Dyhia M Ahmad Haidar A Patrice D. Neutrophil extracellular traps (NET): not only antimicrobial but also modulators of innate and adaptive immunities in inflammatory autoimmune diseases. RMD Open. (2023) 9:e003104. doi: 10.1136/rmdopen-2023-003104, PMID: 37562857 PMC10423839

[124] Wang Y Adair CD Weeks JW Lewis DF Alexander JS. Increased neutrophil-endothelial adhesion induced by placental factors is mediated by platelet-activating factor in preeclampsia. J Soc Gynecol Investig. (1999) 6:136–41. doi: 10.1016/S1071-5576(99)00004-0, PMID: 10376269

[125] Hu Y Li H Yan Y Wang C Wang Y Zhang C. Increased neutrophil activation and plasma DNA levels in patients with pre-eclampsia. Thromb Haemostasis. (2018) 118:2064–2073. doi: 10.1055/s-0038-1675788, PMID: 30453347 PMC6567982

[126] Walsh SW Nugent WH Al Dulaimi M Washington SL Dacha P Strauss JF 3rd. Proteases activate pregnancy neutrophils by a protease-activated receptor 1 pathway: epigenetic implications for preeclampsia. Reprod Sci. (2020) 27:2115–27. doi: 10.1007/s43032-020-00232-4, PMID: 32542542 PMC7529957

[127] Jing Y Shaheen E Drake RR Chen N Gravenstein S Deng Y. Aging is associated with a numerical and functional decline in plasmacytoid dendritic cells, whereas myeloid dendritic cells are relatively unaltered in human peripheral blood. Hum Immunol. (2009) 70:777–84. doi: 10.1016/j.humimm.2009.07.005, PMID: 19596035 PMC5718338

[128] Moldenhauer LM Diener KR Thring DM Brown MP Hayball JD Robertson SA. Cross-presentation of male seminal fluid antigens elicits T cell activation to initiate the female immune response to pregnancy1. J Immunol. (2009) 182:8080–93. doi: 10.4049/jimmunol.0804018, PMID: 19494334

[129] Wang J Tao YM Cheng XY Zhu TF Chen ZF Yao H. Vascular endothelial growth factor affects dendritic cell activity in hypertensive disorders of pregnancy. Mol Med Rep. (2015) 12:3781–6. doi: 10.3892/mmr.2015.3783, PMID: 25975204

[130] Panda B Panda A Ueda I Abrahams VM Norwitz ER Stanic AK. Dendritic cells in the circulation of women with preeclampsia demonstrate a pro-inflammatory bias secondary to dysregulation of TLR receptors. J Reprod Immunol. (2012) 94:210–5. doi: 10.1016/j.jri.2012.01.008, PMID: 22440523

[131] Gierman LM Silva GB Pervaiz Z Rakner JJ Mundal SB Thaning AJ. TLR3 expression by maternal and fetal cells at the maternal-fetal interface in normal and preeclamptic pregnancies. J Leukocyte Biol. (2020) 109:173–83. doi: 10.1002/JLB.3MA0620-728RR, PMID: 32573856

[132] Herrock OT Deer E Amaral LM Campbell N Lemon J Ingram N. B2 cells contribute to hypertension and natural killer cell activation possibly via AT1-AA in response to placental ischemia. Am J Physiol Renal Physiol. (2023) 324:F179–f192. doi: 10.1152/ajprenal.00190.2022, PMID: 36417275 PMC9844978

[133] Salby SB Persson G Pedersen NH Turan G Kimmerslev L Finne KF. Reduced expression of programmed cell death protein 1 on peripheral regulatory B cells in pre-eclampsia – Signs of impaired immune suppression. J Reprod Immunol. (2025) 168:104426. doi: 10.1016/j.jri.2025.104426, PMID: 39823688

[134] Palma AM Hanes MR Marshall JS. Mast cell modulation of B cell responses: an under-appreciated partnership in host defence. Front Immunol. (2021) 12:718499. doi: 10.3389/fimmu.2021.718499, PMID: 34566974 PMC8460918

[135] Wang Y Chen A. Mast cell-derived exosomal miR-181a-5p modulated trophoblast cell viability, migration, and invasion via YY1/MMP-9 axis. J Clin Lab Anal. (2022) 36:e24549. doi: 10.1002/jcla.24549, PMID: 35698293 PMC9280008

[136] Szewczyk G Pyzlak M Klimkiewicz J Smiertka W Miedzińska-Maciejewska M Szukiewicz D. Mast cells and histamine: do they influence placental vascular network and development in preeclampsia? Mediators Inflammation. (2012) 2012:307189. doi: 10.1155/2012/307189, PMID: 22778495 PMC3388381

[137] Pawankar R Okuda M Yssel H Okumura K Ra C. Nasal mast cells in perennial allergic rhinitics exhibit increased expression of the Fc epsilonRI, CD40L, IL-4, and IL-13, and can induce IgE synthesis in B cells. J Clin Invest. (1997) 99:1492–9. doi: 10.1172/JCI119311, PMID: 9119992 PMC507968

[138] Breitling S Hui Z Zabini D Hu Y Hoffmann J Goldenberg NM. The mast cell–B cell axis in lung vascular remodeling and pulmonary hypertension. Am J Physiology-Lung Cell Mol Physiol. (2017) 312:L710–21. doi: 10.1152/ajplung.00311.2016, PMID: 28235950

