# Supplementary material for: Multiple analytical perspectives of mitochondrial genes in the context of preeclampsia: potential diagnostic markers
Source: Front Immunol. 2025 Jul 17;16:1595706. doi: 10.3389/fimmu.2025.1595706 (PMC12310717; doi:10.3389/fimmu.2025.1595706)
Supplement: Supplementary file 1 [file DataSheet1.docx]

**SupplementaryFig.1**
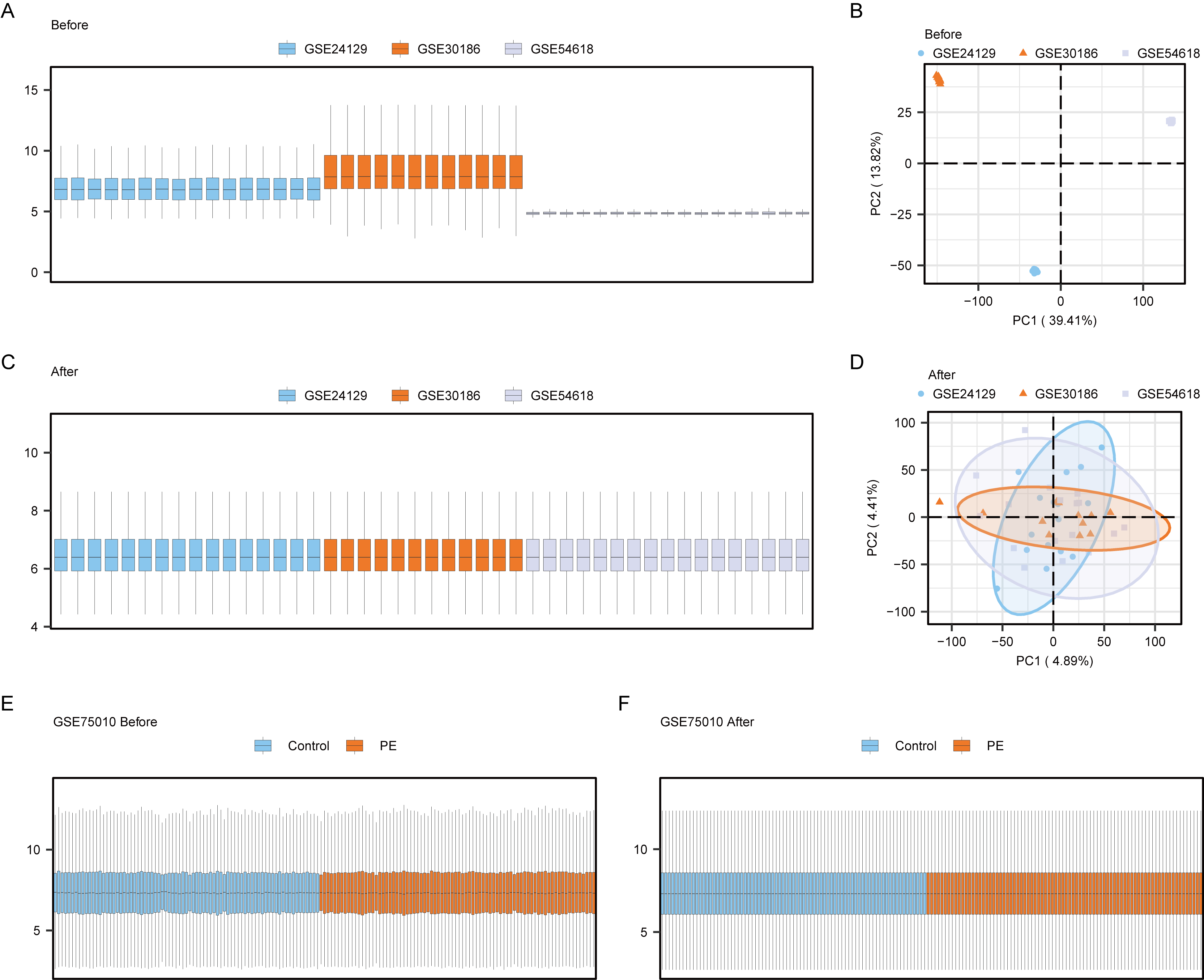
**Supplementary Fig.1 Data set normalization**

### A-B. Boxplot plot (A) and PCA plot (B) of Combined dataset before removing batch effects and normalizing. C-D. Boxplot plot (C) and PCA plot (D) of Combined dataset after removing batch effects and normalizing treatment. E-F. Boxplot plot of dataset GSE75010 before(E) and after(F) normalizing treatment PE, Preeclampsia; PCA, Principal Component Analysis.

**SupplementaryFig.2**


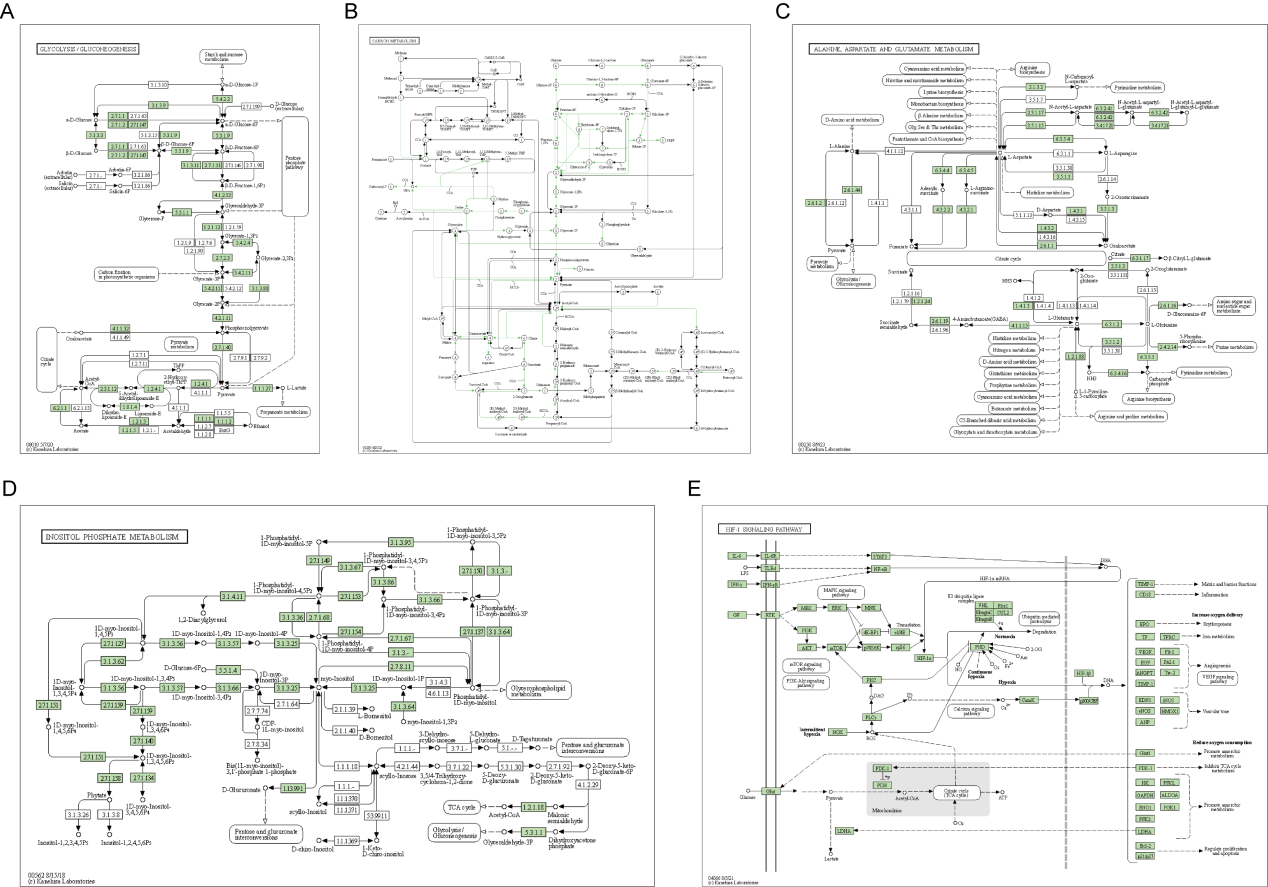


**Supplementary Fig.2 KEGG pathway presentation of MMRDEGs**

1. E. Glycolysis/Gluconeogenesis (A), Carbon metabolism (B), Alanine, aspartate and glutamate metabolism (C), Inositol phosphate metabolism (D), HIF-1 signaling pathway (E). MMRDEGs, Mitochondrial energy metabolism related differentially expressed genes; KEGG, Kyoto Encyclopedia of Genes and Genomes.

**SupplementaryFig.3**


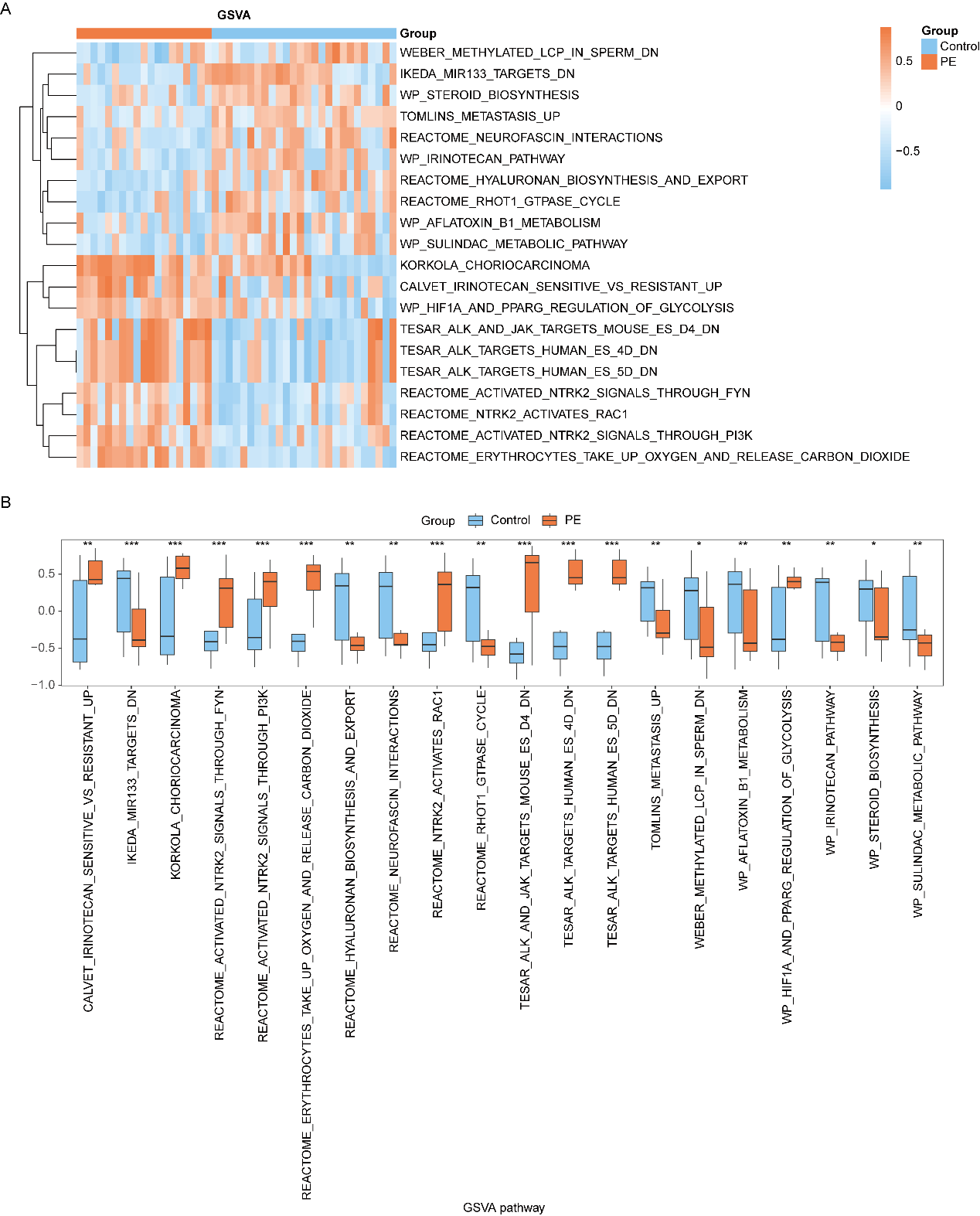
**Supplementary Fig.3 GSVA enrichment analysis between PE cases and Control groups in Combined dataset**

1. B. Heat map (A) and group comparison plot (B) of GSVA enrichment analysis results between PE cases and Control groups in Combined dataset. PE, preeclampsia; GSVA, Gene Set Variation Analysis. The symbol ns is equivalent to *p ≥ 0.05,* and has no statistical significance; The symbol * is equivalent to *p < 0.05*, which is statistically significant; The symbol ** is equivalent to *p < 0.01*, which is highly statistically significant; The symbol *** is equivalent to *p < 0.001* and highly statistically significant.

**SupplementaryFig.4**


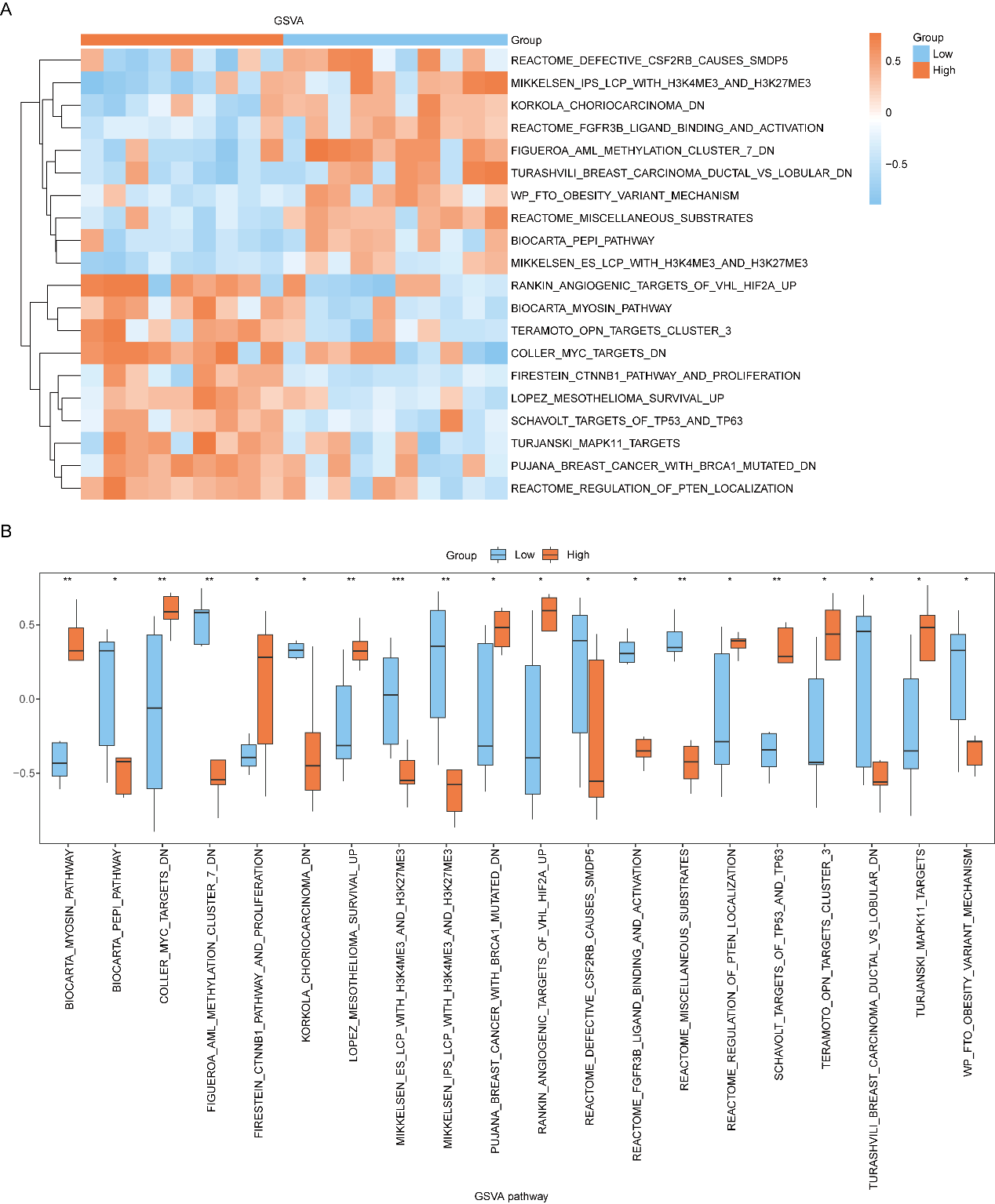
**Supplementary Fig.4 GSVA enrichment analysis between High and Low Risk-score groups in Combined dataset**

1. B. Heat map (A) and group comparison plot (B) of GSVA enrichment analysis results between High and Low Risk-score groups in Combined dataset. MMRDEGs, Mitochondrial energy metabolism related differentially expressed genes; GSVA, Gene Set Variation Analysis. The symbol ns is equivalent to *p ≥ 0.05* and has no statistical significance; The symbol * is equivalent to *p < 0.05*, which is statistically significant; The symbol ** is equivalent to *p < 0.01*, which is highly statistically significant; The symbol *** is equivalent to *p < 0.001* and highly statistically significant.

**SupplementaryFig.5**


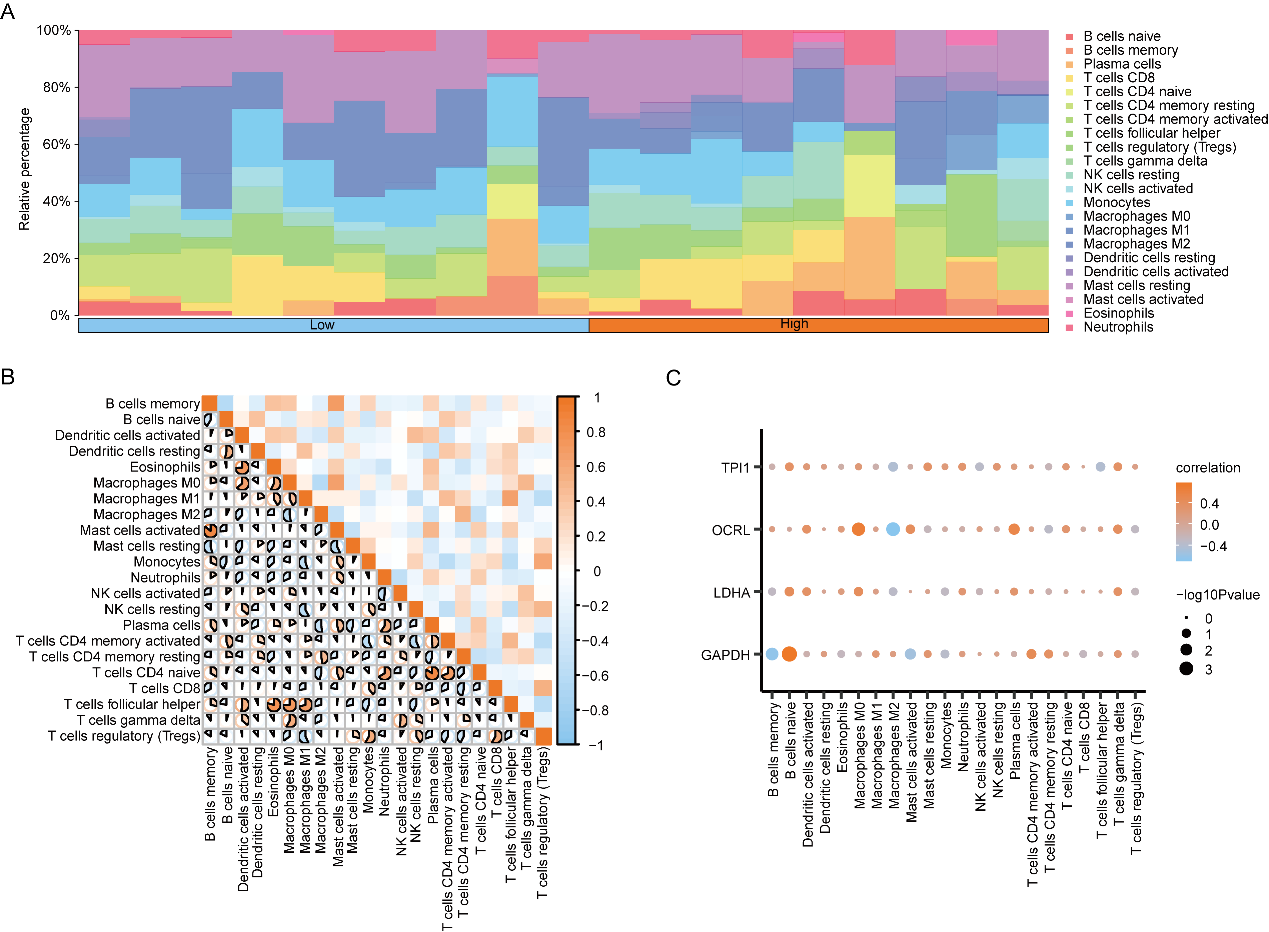
**Supplementary Fig.5 CIBERSORT immune characteristics difference analysis between High and Low Risk-score groups in Combined dataset**

1. The stacked bar chart of CIBERSORT immune infiltration analysis results between the Low/High Risk-score group of Combined dataset data B. Correlation between immune cells with non-zero infiltration abundance in Combined dataset. C. Dot plot of correlation between immune cells with non-zero infiltration abundance and Common MMRDEGs in Combined dataset. Common MMRDEGs, Common Mitochondrial energy metabolism related differentially expressed genes; PE: Preeclampsia.

### Supplementary Table 1. PE Dataset Information list.

|  | GSE24129 | GSE30186 | GSE54618 | GSE75010 |
| --- | --- | --- | --- | --- |
| Platform | GPL6244 | GPL10558 | GPL10558 | GPL6244 |
| Species | Homo sapiens | Homo sapiens | Homo sapiens | Homo sapiens |
| Tissue | placentas | placentas | placentas | placentas |
| Samples in PE cases | 8 | 6 | 5 | 80 |
| Samples in Control group | 8 | 6 | 12 | 77 |
| Reference | Comparative gene expression profiling of placentas from patients with severe pre-eclampsia and unexplained fetal growth restriction | Identification of differential gene expression profiles in placentas from preeclamptic pregnancies versus normal pregnancies by DNA microarrays | Increased glucocerebrosidase expression and activity in preeclamptic placenta | Placental transcriptional and histologic subtypes of normotensive fetal growth restriction are comparable to preeclampsia |

PE, Preeclampsia.

### Supplementary Table 2. List of MMRGs.

| MMRGs | MMRGs | MMRGs | MMRGs | MMRGs | MMRGs |
| --- | --- | --- | --- | --- | --- |
| *ACADL* | *NDUFS3* | *COX17* | *TANGO2* | *BDNF* | *NEU1* |
| *ALDH18A1* | *PKLR* | *HADHB* | *LIAS* | *NR1H4* | *PRDX6* |
| *ADH1B* | *NDUFA8* | *DLD* | *NFATC4* | *CRAT* | *RYR2* |
| *PPAT* | *HADHA* | *ACOX3* | *ETFA* | *NGLY1* | *YWHAZ* |
| *GAPDH* | *PDHX* | *ALDH1L1-AS1* | *AK2* | *DHTKD1* | *ADORA2A* |
| *ALDH2* | *NDUFB3* | *SDHB* | *CYCS* | *GPBAR1* | *COX4I1* |
| *ACSL4* | *ALDH1L1* | *NDUFS1* | *SLC25A20* | *CHCHD10* | *HTR3A* |
| *PPARG* | *CPT1A* | *ALDH3A2* | *SLC25A3* | *PKHD1* | *HUWE1* |
| *CYP2U1* | *CYP4A22* | *LHPP* | *ETFB* | *UCHL1* | *UQCRC2* |
| *PFKP* | *PPARGC1A* | *PPARA* | *FBXL4* | *GSK3B* | *XBP1* |
| *ACSL1* | *CYP4A27P* | *ACSL6* | *COQ9* | *ASS1* | *BSG* |
| *ADH1A* | *ADH1C* | *PPARD* | *PTEN* | *PRKN* | *SIRT5* |
| *GPI* | *EHHADH* | *ACSBG2* | *PDP1* | *ATP7B* | *ATG7* |
| *ALDH3B2* | *NDUFB10* | *NDUFB7* | *UCP2* | *MFN2* | *BAD* |
| *NDUFS6* | *OXCT2* | *ALDH5A1* | *SOD1* | *PAH* | *CKB* |
| *CYP4A22-AS1* | *OXCT1* | *HADH* | *TP53* | *IRS1* | *CLOCK* |
| *PC* | *ACADM* | *NDUFS7* | *TLR2* | *SCN9A* | *EIF2S1* |
| *MDH2* | *ALDH4A1* | *ACSL6-AS1* | *CNR1* | *SOD2* | *FUS* |
| *CYC1* | *CS* | *ALDH1A1* | *SCO2* | *TPI1* | *GDAP1* |
| *PFKFB2* | *PDHB* | *PPA2* | *UCP1* | *BTD* | *IGF2BP2* |
| *ACAT1* | *NDUFA7* | *NDUFA4* | *AK3* | *KCNK9* | *PNPLA6* |
| *CPT1B* | *NDUFA5* | *COX15* | *UCP3* | *VDAC1* | *SHMT1* |
| *ACAA2* | *COX7B* | *PFKM* | *TCF19* | *ATP5F1B* | *SLC25A5* |
| *CYP4A26P* | *CPT1C* | *NDUFC2* | *LDHA* | *OCRL* | *TFAM* |
| *NDUFB4* | *UQCRFS1* | *BPGM* | *EIF4E* | *KRIT1* | *DBT* |
| *ACAA1* | *ACLY* | *NDUFS4* | *RPS6KB1* | *MIPEP* | *EPO* |
| *MDH1B* | *GCDH* | *ADH6* | *KNG1* | *RARS2* | *ESRRG* |
| *NDUFS8* | *PFKL* | *NDUFV3* | *PRKCZ* | *SUMF1* | *ID2* |
| *NDUFAB1* | *IDH3G* | *ACSL3* | *POLG* | *FLVCR2* | *LRPPRC* |
| *ALDH3B1* | *ACOX2* | *ACAT2* | *EIF4EBP1* | *SLC27A1* | *PDK4* |
| *COX5A* | *NDUFA10* | *ALDH16A1* | *ACAD9* | *SZT2* | *PTPA* |
| *ATP4A* | *PFKFB1* | *SDHD* | *CR1* | *MITD1* | *SOAT2* |
| *OGDH* | *GAPDHS* | *NDUFB2* | *KLK4* | *FAM210B* | *UQCRC1* |
| *COX5B* | *ECI1* | *ACADVL* | *TNNT1* | *MT-ND4* | *HTR2B* |
| *PFKP-DT* | *COX10* | *ACSL3-AS1* | *CD200* | *ESR1* | *NDUFAF1* |
| *ACSBG1* | *ALDH8A1* | *NDUFB6* | *OMA1* | *PSEN1* | *NME4* |
| *COX6C* | *ALDH6A1* | *NDUFC1* | *DNAJC19* | *ATP2A2* | *PROK2* |
| *NDUFB9* | *NDUFA11* | *ALDH7A1P2* | *SUCLA2* | *CAT* | *SURF1* |
| *NDUFS2* | *CYP4A44P* | *COX7C* | *GFM1* | *FOXO1* | *BICD2* |
| *ALDH1B1* | *ALDH9A1* | *GALM* | *TUFM* | *NOTCH3* | *DMRT1* |
| *ALDH1A2* | *ADH4* | *ALDH7A1P4* | *AUH* | *ADK* | *IMMT* |
| *NDUFV1* | *NDUFA6* | *PPATP2* | *TSFM* | *APOE* | *INF2* |
| *SDHA* | *MINPP1* | *ALDH7A1* | *HIBCH* | *CBS* | *OLA1* |
| *PPARGC1B* | *AKR1A1* | *ALDH1A3* | *TAFAZZIN* | *CASP7* | *RYR3* |
| *NDUFA9* | *ACO1* | *ACSL5* | *TMEM70* | *EIF2AK3* | *SSBP1* |
| *CYP4A11* | *NDUFV2* | *PPAN-P2RY11* | *MTIF2* | *GLS* | *ATP5PF* |
| *NDUFB11* | *NDUFA13* | *ALDH7A1P3* | *OPA3* | *HSPD1* | *GLRX5* |
| *NDUFA12* | *NDUFA1* | *NDUFB8* | *CENPO* | *PGK1* | *MAVS* |
| *PFKFB4* | *DLAT* | *ALDH1L1-AS2* | *SERAC1* | *SLC25A4* | *NDUFAF3* |
| *IDH1* | *PDC* | *ALDH7A1P1* | *MTIF3* | *STIM1* | *SIRT4* |
| *ACADSB* | *ADPGK* | *ACO2* | *LIX1* | *YWHAE* | *SMPD2* |
| *ATP4B* | *IDH3B* | *OXCT1-AS1* | *C2orf88* | *ADORA2B* | *ALYREF* |
| *ALDH3A1* | *PPATP1* | *COX11* | *NFU1* | *HPRT1* | *COX7A2L* |
| *PAAF1* | *ATP12A* | *OXCT2P1* | *LIPT2* | *HSPA9* | *ACAD10* |
| *ECHS1* | *ECI2* | *NDUFA2* | *FH* | *KCNJ2* | *BOLA3* |
| *ACADS* | *CPT2* | *IDH3A* | *LIPT1* | *PKD2* | *FAHD1* |
| *PPAN* | *AHR* | *NDUFA3* | *IBA57* | *RYR1* | *MRS2* |
| *SDHC* | *MDH1P2* | *NDUFB1* | *SRC* | *VCL* | *SLC25A27* |
| *MDH1* | *CYP4A43P* | *PPA1* | *SIRT3* | *FXN* | *TIMMDC1* |
| *PGM2* | *HMGCL* | *MDH1P1* | *ESRRA* | *HK2* | *ATP5MC1* |
| *PFKFB3* | *POR* | *C1QBP* | *TPK1* | *HTR2A* | *OCIAD1* |
| *NDUFS5* | *ACOX1* | *GFM2* | *AGK* | *IL1B* | *MT-ATP6* |
| *NDUFB5* | *GCK* | *TIGAR* | *MFF* | *INS* | *MT-CO1* |
| *ALDH1L2* | *DLST* | *HTT* | *AIFM1* | *KCNJ5* | *MT-ND1* |

MMRGs, Mitochondrial energy metabolism-related genes.

### SupplementaryTable 3. mRNA primer sequences.

| mRNA | Sequences of the primer |
| --- | --- |
| *OCRL* | 5’-AAGCCTGTTAGCGCCCTCTTC-3’ (forward) |
| *OCRL* | 5’-CACAAACTCCCTCCTGCTGAGT-3’(reverse) |
| *TPI1* | 5’-CTGAGAAGGTTGTTTTCGAGCAGAC-3’ (forward) |
| *TPI1* | 5’-TACCAATGGCCCACACAGGCT-3’(reverse) |
| *LDHA* | 5’-CATGGCCTGTGCCATCAGTATC-3’ (forward) |
| *LDHA* | 5’-TGCCAGAGACAATCTTTGGTGTTC-3’ (reverse) |
| *GAPDH* | 5’-GCACCGTCAAGGCTGAGAAC-3’ (forward) |
| *GAPDH* | 5’-TGGTGAAGACGCCAGTGGA-3’ (reverse) |
| *β-actin* | 5’-GAAGAGCTACGAGCTGCCTGA-3’ (forward) |
| *β-actin* | 5’-CAGACAGCACTGTGTTGGCG-3’ (reverse) |

### Supplementary Table 4. GO and KEGG enrichment analysis results of MMRDEGs.

| ONTOLOGY | ID | Description | GeneRatio | BgRatio | *P* |
| --- | --- | --- | --- | --- | --- |
| BP | GO:0006090 | pyruvate metabolic process | 6/15 | 106/18800 | 1.34 e-10 |
| BP | GO:0006096 | glycolytic process | 5/15 | 81/18800 | 3.8 e-09 |
| BP | GO:0006757 | ATP generation from ADP | 5/15 | 82/18800 | 4.05 e-09 |
| BP | GO:0006091 | generation of precursor metabolites and energy | 6/15 | 494/18800 | 1.31 e-06 |
| BP | GO:0046034 | ATP metabolic process | 5/15 | 273/18800 | 1.66 e-06 |
| CC | GO:0005759 | mitochondrial matrix | 5/16 | 473/19594 | 2.81 e-05 |
| MF | GO:0016620 | oxidoreductase activity, acting on the aldehyde or oxo group of donors, NAD or NADP as acceptor | 4/16 | 38/18410 | 2.76 e-08 |
| MF | GO:0004029 | aldehyde dehydrogenase (NAD+) activity | 2/16 | 15/18410 | 7.39 e-05 |
| MF | GO:0004030 | aldehyde dehydrogenase [NAD(P)+] activity | 2/16 | 16/18410 | 8.44 e-05 |
| MF | GO:0019903 | protein phosphatase binding | 2/16 | 148/18410 | 0.007154 |
| MF | GO:0016646 | oxidoreductase activity, acting on the CH-NH group of donors, NAD or NADP as acceptor | 1/16 | 19/18410 | 0.016392 |
| KEGG | hsa00010 | Glycolysis / Gluconeogenesis | 5/15 | 67/8164 | 9.02 e-08 |
| KEGG | hsa04066 | HIF-1 signaling pathway | 4/15 | 109/8164 | 3.66 e-05 |
| KEGG | hsa01200 | Carbon metabolism | 4/15 | 115/8164 | 4.52 e-05 |
| KEGG | hsa00250 | Alanine, aspartate and glutamate metabolism | 2/15 | 37/8164 | 0.002022 |
| KEGG | hsa00562 | Inositol phosphate metabolism | 2/15 | 73/8164 | 0.00768 |

GO, Gene Ontology; BP, biological process; CC, cellular component; MF, molecular function; KEGG: Kyoto Encyclopedia of Genes and Genomes; MMRDEGs, Mitochondrial energy metabolism related differentially expressed genes.

**Supplementary Table 5. GSEA enrichment analysis results of Combined dataset Control-PE group genes.**

| ID | setSize | enrichmentScore | NES | *P* |
| --- | --- | --- | --- | --- |
| CELL SURFACE INTERACTIONS AT THE VASCULAR WALL | 124 | 0.471741 | 1.890626 | 0.001406 |
| IL6/7 PATHWAY | 45 | 0.539192 | 1.807437 | 0.004815 |
| IL2 SIGNALING PATHWAY | 41 | 0.474468 | 1.556598 | 0.024311 |
| NOTCH SIGNALING PATHWAY | 44 | 0.465621 | 1.552478 | 0.025806 |
| IL9 SIGNALING PATHWAY | 16 | 0.588441 | 1.570026 | 0.031858 |
| VASCULAR SMOOTH MUSCLE CONTRACTION | 99 | 0.31003 | 1.34865 | 0.039474 |

GSEA, Gene Set Enrichment Analysis; PE, Preeclampsia.

**Supplementary Table 6. GSVA enrichment analysis results of Combined dataset Control-PE group genes.**

|  | logFC | AveExpr | t | *P* |
| --- | --- | --- | --- | --- |
| MIR133 TARGETS DN | 0.46171 | 0.0306 | 3.6741 | 0.00057 |
| HYALURONAN BIOSYNTHESIS AND EXPORT | 0.45345 | 0.07697 | 3.7369 | 0.000469 |
| RHOT1 GTPASE CYCLE | 0.43051 | 0.11792 | 3.42131 | 0.001229 |
| NEUROFASCIN INTERACTIONS | 0.42503 | 0.04473 | 3.51325 | 0.000932 |
| IRINOTECAN PATHWAY | 0.41309 | 0.0919 | 3.41926 | 0.001237 |
| AFLATOXIN B1 METABOLISM | 0.39016 | 0.009256 | 2.93596 | 0.004962 |
| SULINDAC METABOLIC PATHWAY | 0.36684 | 0.15456 | 2.94709 | 0.004813 |
| METHYLATED LCP IN SPERM DN | 0.36048 | 0.09008 | 2.62172 | 0.01148 |
| METASTASIS UP | 0.35716 | 0.037787 | 3.6838 | 0.000553 |
| STEROID BIOSYNTHESIS | 0.34723 | 0.04532 | 3.08743 | 0.00325 |
| ACTIVATED NTRK2 SIGNALS THROUGH FYN | 0.464792 | 0.08448 | 3.916066 | 0.000266 |
| NTRK2 ACTIVATES RAC1 | 0.47932 | 0.08652 | 3.802727 | 0.000381 |
| HIF1A AND PPARG REGULATION OF GLYCOLYSIS | 0.480345 | 0.018004 | 4.001881 | 0.000202 |
| ACTIVATED NTRK2 SIGNALS THROUGH PI3K | 0.484743 | 0.01088 | 4.180783 | 0.000113 |
| IRINOTECAN SENSITIVE VS RESISTANT UP | 0.53309 | 0.055218 | 3.644371 | 0.000624 |
| CHORIOCARCINOMA | 0.566571 | 0.172127 | 3.95932 | 0.000232 |
| ERYTHROCYTES TAKE UP OXYGEN AND RELEASE CARBON DIOXIDE | 0.654175 | 0.01115 | 5.899596 | 2.87 e-07 |
| ALK TARGETS HUMAN ES 4D DN | 0.706038 | 0.0151 | 4.988842 | 7.32 e-06 |
| ALK TARGETS HUMAN ES 5D DN | 0.706038 | 0.0151 | 4.988842 | 7.32 e-06 |
| ALK AND JAK TARGETS MOUSE ES D4 DN | 0.76107 | 0.07389 | 4.811497 | 1.35 e-05 |

GSVA, Gene Set Variation Analysis; PE, Preeclampsia.

**Supplementary Table 7. clinical characteristics of patients in the study**

|  | Control group（n=20） | PE cases  (n=20) | *t* value | *P* value |
| --- | --- | --- | --- | --- |
| Age(years) | 33.00±3.81 | 33.90±4.55 | 0.6335 | 0.5302 |
| Gravida(times) | 2.35±1.50 | 2.40±1.70 | 0.1995 | 0.8430 |
| Para(times) | 1.70±0.73 | 1.60±0.82 | 0.2043 | 0.8392 |
| Body mass index(BMI)(kg/m2) | 28.99±3.02 | 29.51±3.18 | 0.5665 | 0.5744 |
| Systolic pressure(mmHg) | 121.70±10.24 | 154.85±15.51 | 8.271 | <0.0001^****^ |
| Diastolic pressure(mmHg) | 76.95±7.05 | 97.75±14.36 | 5.506 | <0.0001^****^ |
| Mean arterial pressure(MAP) | 91.87±6.65 | 116.78±12.75 | 7.505 | <0.0001^****^ |
| Gestational week | 38.20±1.54 | 35.05±3.39 | 3.778 | <0.0005^***^ |
| Altanine aminotransferase(ALT) | 11.84±5.30 | 22.69±23.96 | 2.090 | 0.043^*^ |
| Aspartate aminotransferase(AST) | 14.70±3.92 | 21.16±17.26 | 1.735 | 0.0908 |
| ALT/AST | 0.80±0.27 | 1.01±0.52 | 1.770 | 0.0848 |
| Alkaline phosphatase(ALP) | 125.99±43.45 | 133.29±46.74 | 0.4398 | 0.6626 |
| Uric acid | 278.89±55.60 | 443.89±111.43 | 5.574 | <0.0001^****^ |
| Platelet | 211.45±45.11 | 200.75±68.69 | 1.003 | 0.3221 |
| Hemoglobin(Hb) | 118.40±10.17 | 123.20±12.58 | 0.9732 | 0.3366 |
| Albumin | 35.22±1.80 | 31.35±4.09 | 4.030 | 0.0003^***^ |
| Creatinine | 64.93±6.49 | 68.82±14.25 | 1.122 | 0.2689 |

PE, Preeclampsia. The symbol * is equivalent to *p* < 0.05 and is highly statistically significant; The symbol *** is equivalent to *p* < 0.001 and is highly statistically significant; The symbol **** is equivalent to *p* < 0.0001 and is highly statistically significant.

**Supplementary Table 8. GSEA enrichment analysis results of Combined dataset Low-High Riskscore group genes.**

| ID | setSize | enrichmentScore | NES | *P* |
| --- | --- | --- | --- | --- |
| ELECTRON TRANSPORT CHAIN OXPHOS SYSTEM IN MITOCHONDRIA | 64 | 0.66226 | 2.466736 | 0.00159 |
| ENERGY METABOLISM | 46 | 0.486872 | 1.711386 | 0.008052 |
| IL6 PATHWAY | 20 | 0.593013 | 1.74051 | 0.011765 |
| IL5 SIGNALING PATHWAY | 38 | 0.48862 | 1.651983 | 0.013072 |
| IL7 SIGNALING PATHWAY | 25 | 0.483819 | 1.495271 | 0.040584 |
| THE CITRIC ACID TCA CYCLE AND RESPIRATORY ELECTRON TRANSPORT | 127 | 0.577311 | 2.415895 | 0.001443 |

GSEA, Gene Set Enrichment Analysis.

**Supplementary Table 9. GSVA enrichment analysis results of Combined dataset Low-High Riskscore group genes.**

|  | logFC | AveExpr | t | *P* |
| --- | --- | --- | --- | --- |
| MYOSIN PATHWAY | 0.56837 | 0.01782 | 3.68593 | 0.00106 |
| ANGIOGENIC TARGETS OF VHL HIF2A UP | 0.56559 | 0.018056 | 2.55504 | 0.016848 |
| MYC TARGETS DN | 0.56325 | 0.177881 | 2.59998 | 0.0152 |
| CTNNB1 PATHWAY AND PROLIFERATION | 0.5228 | 0.13563 | 3.8301 | 0.000732 |
| TERAMOTO OPN TARGETS CLUSTER 3 | 0.52018 | 0.023522 | 3.03645 | 0.005403 |
| TARGETS OF TP53 AND TP63 | 0.51502 | 0.02382 | 3.58473 | 0.001374 |
| REGULATION OF PTEN LOCALIZATION | 0.51402 | 0.128541 | 3.21493 | 0.003485 |
| MAPK11 TARGETS | 0.50672 | 0.019196 | 2.66281 | 0.013147 |
| MESOTHELIOMA SURVIVAL UP | 0.50472 | 0.044765 | 3.60537 | 0.001303 |
| BREAST CANCER WITH BRCA1 MUTATED DN | 0.49331 | 0.138277 | 2.7908 | 0.009742 |
| PEPI PATHWAY | 0.471466 | 0.16218 | 2.972076 | 0.006316 |
| DEFECTIVE CSF2RB CAUSES SMDP5 | 0.472303 | 0.04822 | 2.369865 | 0.025537 |
| FTO OBESITY VARIANT MECHANISM | 0.472606 | 0.04778 | 3.217338 | 0.003464 |
| FGFR3B LIGAND BINDING AND ACTIVATION | 0.519242 | 0.03754 | 3.599427 | 0.001323 |
| ES LCP WITH H3K4ME3 AND H3K27ME3 | 0.520521 | 0.24503 | 4.535695 | 0.000116 |
| CHORIOCARCINOMA DN | 0.545806 | 0.02504 | 3.339513 | 0.002555 |
| BREAST CARCINOMA DUCTAL VS LOBULAR DN | 0.578262 | 0.13376 | 3.064848 | 0.005042 |
| MISCELLANEOUS SUBSTRATES | 0.649187 | 0.00605 | 4.51058 | 0.000124 |
| IPS LCP WITH H3K4ME3 AND H3K27ME3 | 0.676623 | 0.08169 | 3.627009 | 0.001233 |
| AML METHYLATION CLUSTER 7 DN | 0.700087 | 0.024266 | 3.609487 | 0.00129 |

GSVA, Gene Set Variation Analysis.

### Supplementary Table 10. mRNA-RBP interaction network nodes.

| mRNA | RBP |  | mRNA | RBP |
| --- | --- | --- | --- | --- |
| *GAPDH* | ALYREF |  | *OCRL* | FIP1L1 |
| *GAPDH* | CENPC |  | *OCRL* | FMR1 |
| *GAPDH* | EIF3B |  | *OCRL* | FTO |
| *GAPDH* | ENO1 |  | *OCRL* | FUBP1 |
| *GAPDH* | METTL1 |  | *OCRL* | G3BP1 |
| *GAPDH* | SCAF8 |  | *OCRL* | GRSF1 |
| *GAPDH* | SNRPA |  | *OCRL* | HNRNPK |
| *GAPDH* | TARBP2 |  | *OCRL* | HNRNPU |
| *LDHA* | CAPRIN1 |  | *OCRL* | MSI2 |
| *LDHA* | DDX21 |  | *OCRL* | MTDH |
| *LDHA* | EIF4A3 |  | *OCRL* | NCBP3 |
| *LDHA* | EIF4G2 |  | *OCRL* | NUDT21 |
| *LDHA* | G3BP1 |  | *OCRL* | PRKDC |
| *LDHA* | HNRNPC |  | *OCRL* | PRPF4 |
| *LDHA* | NPM1 |  | *OCRL* | PRPF8 |
| *LDHA* | YBX1 |  | *OCRL* | PUM1 |
| *OCRL* | AQR |  | *OCRL* | RBFOX2 |
| *OCRL* | ATXN2 |  | *OCRL* | RC3H1 |
| *OCRL* | BCLAF1 |  | *OCRL* | SCAF4 |
| *OCRL* | CAPRIN1 |  | *OCRL* | SCAF8 |
| *OCRL* | CPSF2 |  | *OCRL* | TENT4B |
| *OCRL* | CPSF6 |  | *OCRL* | YTHDC1 |
| *OCRL* | CPSF7 |  | *OCRL* | YTHDF3 |
| *OCRL* | CSTF2 |  | *TPI1* | ALYREF |
| *OCRL* | CSTF2T |  | *TPI1* | CENPC |
| *OCRL* | CTCF |  | *TPI1* | EIF4A3 |
| *OCRL* | DDX3X |  | *TPI1* | RC3H1 |
| *OCRL* | DHX36 |  | *TPI1* | RTCB |
| *OCRL* | FAM120A |  | *TPI1* | SRSF9 |

RBP, RNA binding protein.

### Supplementary Table 11. mRNA-TF interaction network nodes.

| mRNA | TF |  | mRNA | TF |
| --- | --- | --- | --- | --- |
| *OCRL* | BRD4 |  | *GAPDH* | ETS1 |
| *OCRL* | CTCF |  | *GAPDH* | ETV1 |
| *OCRL* | EP300 |  | *GAPDH* | FLI1 |
| *OCRL* | ERG |  | *GAPDH* | FOSL2 |
| *OCRL* | FLI1 |  | *GAPDH* | GABPA |
| *OCRL* | GABPA |  | *GAPDH* | HDAC1 |
| *OCRL* | MAZ |  | *GAPDH* | JUN |
| *OCRL* | MAZ |  | *GAPDH* | JUNB |
| *OCRL* | EP300 |  | *GAPDH* | JUND |
| *GAPDH* | BRD4 |  | *GAPDH* | MAX |
| *GAPDH* | CDK9 |  | *GAPDH* | MAZ |
| *GAPDH* | CREBBP |  | *GAPDH* | MXI1 |
| *GAPDH* | CTCF |  | *GAPDH* | NFE2 |
| *GAPDH* | E2F1 |  | *GAPDH* | NFIC |
| *GAPDH* | E2F6 |  | *GAPDH* | NR2F2 |
| *GAPDH* | EGR1 |  | *GAPDH* | SPI1 |
| *GAPDH* | ELK4 |  | *GAPDH* | TBP |
| *GAPDH* | EP300 |  | *TPI1* | BRD2 |
| *GAPDH* | ERG |  | *TPI1* | EGR1 |
| *GAPDH* | ESR1 |  |  |  |

TF, Transcription factors.

### Supplementary Table 12. mRNA-Drug interaction network nodes.

| mRNA | Drug | mRNA | Drug |
| --- | --- | --- | --- |
| *OCRL* | bisphenol A | *LDHA* | Cadmium Chloride |
| *OCRL* | cobaltous chloride | *LDHA* | Calcitriol |
| *OCRL* | Copper | *LDHA* | cobaltous chloride |
| *OCRL* | Hydrogen Peroxide | *LDHA* | Fenretinide |
| *OCRL* | nickel chloride | *LDHA* | (+)-JQ1 compound |
| *OCRL* | Oxygen | *LDHA* | methylmercuric chloride |
| *OCRL* | sodium arsenite | *LDHA* | Oxygen |
| *OCRL* | Tretinoin | *LDHA* | Potassium Dichromate |
| *LDHA* | 2, 3 ', 4, 4 ', 5 - pentachlorobiphenyl | *LDHA* | sodium arsenite |
| *LDHA* | 3-methyladenine | *LDHA* | trichostatin A |
| *LDHA* | 4-phenylbutyric acid | *LDHA* | Valproic Acid |
| *LDHA* | Acetaminophen | *TPI1* | Cyclosporine |
| *LDHA* | Acetylcysteine | *TPI1* | Quercetin |
| *LDHA* | bisphenol A | *TPI1* | sodium arsenite |
| *LDHA* | Cadmium | *TPI1* | Valproic Acid |
